# HsfA1a confers pollen thermotolerance through upregulating antioxidant capacity, protein repair, and degradation in *Solanum lycopersicum* L.

**DOI:** 10.1093/hr/uhac163

**Published:** 2022-07-22

**Authors:** Dong-Ling Xie, Hua-Min Huang, Can-Yu Zhou, Chen-Xu Liu, Mukesh Kumar Kanwar, Zhen-Yu Qi, Jie Zhou

**Affiliations:** Department of Horticulture, Zhejiang Provincial Key Laboratory of Horticultural Plant Integrative Biology, Zhejiang University, Yuhangtang Road 866, Hangzhou, 310058, China; Department of Horticulture, Zhejiang Provincial Key Laboratory of Horticultural Plant Integrative Biology, Zhejiang University, Yuhangtang Road 866, Hangzhou, 310058, China; Department of Horticulture, Zhejiang Provincial Key Laboratory of Horticultural Plant Integrative Biology, Zhejiang University, Yuhangtang Road 866, Hangzhou, 310058, China; Department of Horticulture, Zhejiang Provincial Key Laboratory of Horticultural Plant Integrative Biology, Zhejiang University, Yuhangtang Road 866, Hangzhou, 310058, China; Department of Horticulture, Zhejiang Provincial Key Laboratory of Horticultural Plant Integrative Biology, Zhejiang University, Yuhangtang Road 866, Hangzhou, 310058, China; Hainan Institute, Zhejiang University, Sanya, China; Agricultural Experiment Station, Zhejiang University, Hangzhou 310058, China; Department of Horticulture, Zhejiang Provincial Key Laboratory of Horticultural Plant Integrative Biology, Zhejiang University, Yuhangtang Road 866, Hangzhou, 310058, China; Hainan Institute, Zhejiang University, Sanya, China; Key Laboratory of Horticultural Plants Growth, Development and Quality Improvement, Agricultural Ministry of China, Yuhangtang Road 866, Hangzhou 310058, China; Shandong (Linyi) Institute of Modern Agriculture, Zhejiang University, Linyi 276000, China

## Abstract

The heat shock transcription factors (Hsfs) play critical roles in plant responses to abiotic stresses. However, the mechanism of Hsfs in the regulation of pollen thermotolerance and their specific biological functions and signaling remain unclear. Herein, we demonstrate that HsfA1a played a key role in tomato pollen thermotolerance. Pollen thermotolerance was reduced in *hsfA1a* mutants but was increased by *hsfA1a* overexpression, based on pollen viability and germination. Analyzing the whole transcriptome by RNA-seq data, we found that HsfA1a mainly regulated the genes involved in oxidative stress protection, protein homeostasis regulation and protein modification, as well as the response to biological stress in anthers under heat stress. The accumulation of reactive oxygen species in anthers was enhanced in *hsfA1a* mutants but decreased in *HsfA1a*-overexpressing lines. Furthermore, HsfA1a bound to the promoter region of genes involved in redox regulation (*Cu/Zn-SOD*, *GST8*, and *MDAR1*), protein repair (*HSP17.6A*, *HSP70-2*, *HSP90-2*, and *HSP101*) and degradation (*UBP5*, *UBP18*, *RPN10a*, and *ATG10*) and regulated the expression of these genes in tomato anthers under heat stress. Our findings suggest that HsfA1a maintains pollen thermotolerance and cellular homeostasis by enhancing antioxidant capacity and protein repair and degradation, ultimately improving pollen viability and fertility.

## Highlights

HsfA1a promotes tomato pollen vigor and germination rate under heat stress by transcriptionally regulating the genes involved in antioxidant capacity, protein repair, and degradation capacity in tomato anthers.

## Introduction

Global warming has led to frequent extreme weather, causing huge agricultural production losses every year. The male reproductive organs of plants are particularly sensitive to temperature changes. Heat stress could cause a series of effects on plant pollen in terms of cell structure, physiological metabolism, and molecular mechanism, and ultimately inhibit pollen germination and pollen tube elongation, reduce fruit setting rate, and lead to a decline in yield and quality [1, 2]. Therefore, it is crucial to understand the regulatory mechanisms of pollen thermotolerance in order to alleviate stress damage, carry out germplasm innovation, and maintain food security.

To counteract the damage caused by heat stress and maintain cellular homeostasis, pollen activates a heat stress response (HSR) depending on the intensity and duration of high temperature [1]. The basis of the HSR is a network of heat stress transcription factors (Hsfs) that combine with heat stress elements (HSEs) to induce the expression of downstream genes [3]. Hsfs act as key elements of signal transduction chains and are also the intersection or node of multiple pathways, such as antioxidant processes, hormone regulation, protein quality control (PQC), and metabolic regulation [3]. The Hsf family has 24 members in tomato and 21 in *Arabidopsis* [3, 4]. According to the characteristics of the oligomerization domain, Hsf genes can be divided into three types: A, B, and C [5]. Class A Hsfs (HsfAs) have the function of a transcription activator, which is mediated by a short activation peptide motif (AHA motif). Studies have demonstrated that HsfAs are extensively involved in pollen development and plant responses to heat and other stress conditions. In *Arabidopsis*, knockout of the *HsfA5* homolog gene *AtREN1* results in a defective HSR, leading to a high percentage of abnormal pollen grains [6]. The HSR in tomato pollen is mainly mediated by HsfA1 and its coactivator HsfA2, which in turn regulates the expression of heat shock proteins (HSPs) in stamens and promotes pollen germination [4]. Transcriptome analysis revealed that tomato *HsfA2*, *HsfA3*, *HsfB1*, and *HsfB2b* of the Hsf family are upregulated after heat stress exposure in developing and mature pollen, whereas *HsfA4c* and *HsfA5* remain unaffected [7]. In tomato, HsfA2 has been found in all developmental stages after heat stress. Meanwhile, many other Hsfs appear to have stage-specific responses towards heat stress. *HsfB1* occurs in post-meiotic pollen and mature pollen, while *HsfB2b* is present in pollen in tetrads and during post-meiosis. *HsfA3* and *HsfA7* are much more stage-specific and are expressed in tetrads and the post-meiotic stage, respectively [7]. *HSFC1b* is also responsive to high temperature, and heterologous expression of the *Festuca arundinacea FaHsfC1b* gene can improve *Arabidopsis* survival under heat stress [8]. These studies all indicate that Hsfs play crucial roles in pollen development and heat tolerance; however, the specific biological functions and signaling networks of Hsfs are still largely unknown.

Oxidative stress induced by environmental stimuli leads to the overproduction of reactive oxygen species (ROS), which often affects metabolic homeostasis, disrupting protein stability and membrane fluidity and ultimately causing pollen abortion [1]. Plants have a complex redox regulation system to detoxify ROS and protect anther and pollen development under stresses [9, 10]. Different antioxidant enzymes have different functions in response to high temperature in pollen and anthers. In rice anthers, the activities of superoxide dismutase (SOD), peroxidase (POD), and catalase (CAT) were significantly reduced, while ascorbate peroxidase was relatively stable [11]. Among various SOD enzymes, Cu/Zn-SOD is identified as the central component in regulating total SOD activity in rice anthers [12]. Previous studies have demonstrated the links between antioxidant enzymes and Hsfs in redox regulation [13, 14]. PuHSFA4a increased glutathione S-transferase (GST) activity by binding to the promoter of *PuGSTU17* in *Populus ussuriensis* to reduce ROS acumination and promote excess Zn tolerance [13]. Heterologous expression of *CaHsfA1d* in *Arabidopsis* enhanced the expression of Hsfs and the antioxidant gene *AtGSTU5*, thereby maintaining H_2_O_2_ homeostasis and improving plant thermotolerance [14]. However, Hsf-mediated redox regulation in pollen under heat stress is largely unknown.

Cellular PQC is one of the most essential biological processes under stresses. Stress-induced misfolded, damaged, and truncated proteins are rescued or degraded by PQC [15, 16]. HSP genes are widely studied heat stress-inducible genes and target genes of Hsfs, which play key roles in PQC. HSPs, such as HSP70 and HSP90, can bind to denatured proteins with high affinity, thereby stabilizing, resolubilizing, and refolding denatured proteins [17]. Maintaining the functional conformation of target proteins and preventing denatured protein aggregation contributes to cellular homeostasis under stresses [9]. In addition, small HSPs (sHSPs) show more accumulation in anthers of heat-tolerant genotypes of rice under heat stress, leading to a protective effect on anther proteins at high temperature [1, 18]. Moreover, HSPs also accumulate in unstressed pollen at early stages of development, which can enhance the resistance of pollen to abiotic stresses during development [4].

**Figure 1:**
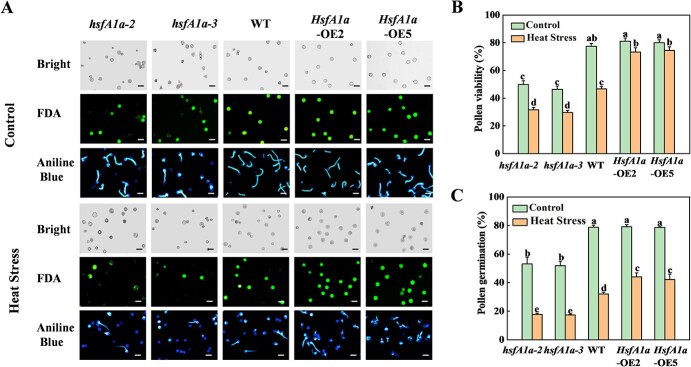
Mutation and overexpression (OE) of *HsfA1a* in tomato affect pollen viability and germination in *Solanum lycopersicum* (tomato) plant under control conditions and heat stress. (A) FDA staining and aniline blue staining of mature pollen grains of *hsfA1a* mutants, WT, and *HsfA1a*-OE plants under the control condition or heat stress. Scale bar: 75 μm. (B, C) Mature pollen viability (B) and germination (C) in *hsfA1a*, WT, and *HsfA1a*-OE plants under the control condition or heat stress. According to Tukey’s test, the same letter indicates that the difference is not significant at *P* < .05. For qualitative analysis, pollen gains were isolated from >30 individual flowers for each genotype and treatment. *hsfA1a*-2 and *hsfA1a*-3, two lines of *hsfA1a* mutants; *HsfA1a*-OE2 and *HsfA1a*-OE5, two lines of *HsfA1a* OE plants.

**Figure 2:**
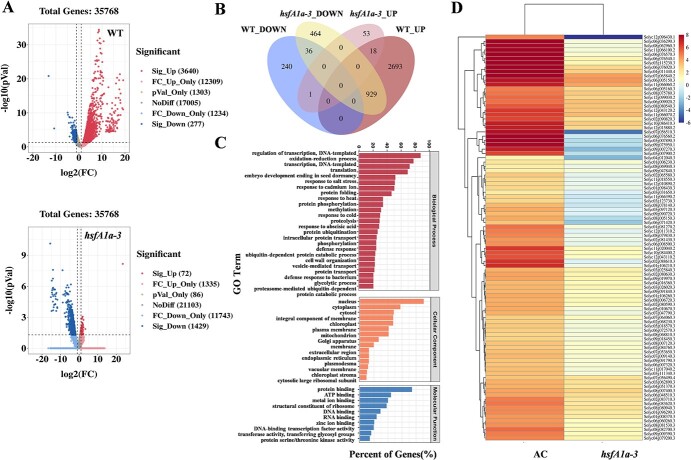
DEGs in WT and *hsfA1a* mutant anthers under heat stress. (A) Volcano plot of DEGs. Red and blue dots represent up- and downregulated genes, respectively, in anthers of WT and *hsfA1a* mutants after heat stress. (B) Venn diagram showing the number of genes significantly changed under heat stress in WT and *hsfA1a* anthers. (C) Classification of heat-induced genes in anthers of WT and *hsfA1a* mutants by using Gene Ontology (GO) functional annotation from Venn diagrams. (D) Heat map of DEGs for antioxidant, protein protection, and degradation-related genes in anthers of WT and *hsfA1a* mutants after heat stress. The color scale represents the different log_2_ (FPKM) values of DEGs. *hsfA1a*-3, one line of *hsfA1a* mutant.

In addition to protein repair, protein degradation mechanisms are actively involved in plant responses to abiotic stresses. The ubiquitin–proteasome system (UPS) and autophagy are two critical pathways for protein degradation [18–20]. The UPS utilizes the protease activity of the 26S proteasome to degrade target proteins, which are ubiquitinated by ubiquitin-activating enzyme (E1), ubiquitin-conjugating enzyme (E2), and the ubiquitin–protein ligase (E3) (E1–E2–E3) conjugated cascade [21]. Meanwhile, the reaction to remove the attached ubiquitin is also essential for the UPS, which is catalyzed by deubiquitinases (DUBs) [22]. Autophagy utilizes the intracellular vesicle transport system to degrade denatured proteins. Damaged organelles or protein aggregates are phagocytosed by double-membrane autophagosomes in the cytoplasm and subsequently transported into vacuoles, where they are broken down by proteases and hydrolases [20]. Due to the drastic changes in energy and matter during anther/pollen development, many studies have shown that these two pathways are involved in the development of anthers and pollens [1, 23]. Mutation of *rpt2a* encoding the 26S proteasomal subunit in *Arabidopsis* showed impaired pollen development and delayed flowering [24]. The ubiquitin-specific proteases AtUBP3/4 in *Arabidopsis* are involved in pollen tube development, and lack of them results in defects in pollen germination [25]. Furthermore, disruption of *autophagy-related gene 6* (*ATG6*) in *Arabidopsis* resulted in defective pollen germination [26]. *ATG2*-, *ATG5*-, and *ATG7*-RNAi lines of *Nicotiana tabacum* exhibit reduced pollen germination [23]. Rice *atg7* mutants exhibit limited anther dehiscence, abnormal autophagosome formation, and reduced rice pollen numbers and fertility [27]. Recent studies have found that protein degradation is not only involved in pollen development, but is also involved in pollen responses to abiotic stresses such as extreme temperature [10, 17]. The membrane-bound E3 ligase BnTR1 can enhance pollen viability to increase rice yield under heat stress [28]. During anther development in *Arabidopsis*, heat stress promotes autophagy and autolysosome formation in wild-type (WT) anther wall cells and microspores, whereas abnormal tapetum degradation, reduced pollen grains, and defective anther dehiscence have been identified in *atg5* mutants after heat stress [29].

The HsfA1 subfamily is defined as the main regulatory factor in response to high-temperature stress. The four HsfA1s AtHsfA1a, b, d, and e in *Arabidopsis* are functionally redundant [3, 30]. In tomato, HsfA1a acts as a major regulator in response to heat stress and cannot be replaced by any other HsfAs [4]. In this study, we created tomato *HsfA1a*-overexpressing and knockout plants to study the HsfA1a-mediated thermotolerance mechanism in pollen. Transcriptome analysis results show that HsfA1a-induced gene expression is involved in the antioxidant system, the protein repair system, and the protein degradation and regeneration system. Moreover, HsfA1a can scavenge ROS accumulation and transcriptionally regulate the abundance of genes that are involved in redox regulation, HSPs, the UPS and autophagy in tomato anthers under heat stress. Therefore, this study provides important information for the regulatory networks and potential applications of HsfA1a in tomato pollen thermotolerance.

## Results

### HsfA1a was essential for pollen viability in tomato under heat stress

To investigate the role of HsfA1a in tomato pollen under heat stress, we tested pollen vigor and germination ability in two lines of *hsfA1a* mutants and *HsfA1a*-overexpressing (*HsfA1a*-OE) plants under heat stress. As shown in Fig. 1, pollen vigor and pollen germination were reduced in *hsfA1a* mutants under normal conditions, but was similar in *HsfA1a*-OE plants compared with WT as revealed by fluorescein diacetate (FDA) staining and aniline blue staining. Importantly, pollen vigor was reduced by 32.3 and 36.7% in *hsfA1a-2* and *hsfA1a-3* mutants, while it was increased by 48.3 and 59.2% in two lines of *HsfA1a*-OE plants under heat stress, respectively (Fig. 1). Similarly, pollen germination was 45.2 and 46.1% lower in *hsfA1a-2* and *hsfA1a-3* mutants, while it was 37.4 and 32.5% higher in *HsfA1a*-OE2 and *HsfA1a*-OE5 plants in WT under high temperature (Fig. 1). These assays suggest that HsfA1a is closely involved in the regulation of pollen thermotolerance in tomato.

### Genome-wide identification of HsfA1a-dependent heat-response genes in anthers

To analyze the global transcriptional alterations caused by HsfA1a in anthers, we selected WT and *hsfA1a-3* mutants for parametric absolute quantitative transcriptome sequencing. Anther samples were obtained from WT and *hsfA1a-3* mutant plants either kept at 25°C (control) or treated at 39°C (heat stress) for 3 hours. The sequencing data statistics are listed in Supplementary Data Table S1, and principal component analysis (PCA) of genes in WT and *hsfA1a* mutant anthers showed that the samples were segregated along PC1 with and without heat stress (Supplementary Data Fig. S2A). Transcriptome analysis revealed that 3640 genes were significantly upregulated in anthers of WT plants after heat stress, compared with only 72 in *hsfA1a* mutants (Fig. 2A). However, 277 genes were downregulated in anthers of WT and 1429 genes were downregulated in the *hsfA1a* mutants after heat stress (Fig. 2A). Results showed that loss of function of tomato HsfA1a has a serious influence on the HSR network and gene expression in anthers. To further study the function of HsfA1a in anthers under heat stress, we performed a crossover analysis of genes that were significantly changed in WT and *hsfA1a* mutant plants after being exposed to heat stress. It was found that 3622 genes in WT were upregulated, of which 929 were downregulated in *hsfA1a* mutants (Fig. 2B). These results suggested that the loss of HsfA1a function affected most heat-responsive gene expression in tomato anthers. Gene Ontology (GO) functional annotation was implemented for 3622 differentially expressed genes (DEGs), and the significantly enriched GO terms (*Q* < 0.05) are shown in Supplementary Data Table S2; the results suggested that these DEGs were involved in multiple cellular functional processes. For instance, in biological processes, DEGs were significantly enriched in transcriptional translation, oxidation–reduction process, protein folding modification, and degradation and stress response (Fig. 2C); in terms of cellular components, DEGs were found in the nucleus, cell membrane, and plastid (Fig. 2C). In the molecular function category, DEGs were significantly enriched in the process of protein binding, ATP binding, and metal ion binding (Fig. 2C). The functional classification of DEGs indicated that the response process of tomato anthers to heat stress mainly focused on redox regulation and protein protection and degradation. Meanwhile, through KEGG pathway analysis, we found that the mitogen-activated protein kinase (MAPK) signaling pathway and phytohormones are mainly involved in the process of signal transduction; ubiquitin-mediated proteolysis and proteasome and protein export are related to the process of protein folding, classification, and degradation; and the process of transport and catabolism mainly involves phagosomes, endocytosis, and peroxisomes (Supplementary Data Table S3, Fig. 2C).

Taken together, these findings showed that HsfA1a-dependent heat-response genes in anthers were mainly involved in the redox process and protein protection and degradation process. Thus, the 94 DEGs involved in these processes were selected with | log_2_(FPKM) | >1 and *P* < 0.05 (where FPKM is fragments per kilobase of transcript per million mapped reads), including 25 antioxidant enzyme-related genes, 28 heat shock-related genes, 22 ubiquitin-related genes, 11 proteasome-related genes, and 8 ATGs, to further study the function of HsfA1a in transcriptional regulation of heat-response genes in tomato anthers (Fig. 2D).

### HsfA1a alleviated reactive oxygen species accumulation by transcriptionally regulating expression of antioxidant enzymes in anthers under heat stress

**Figure 3:**
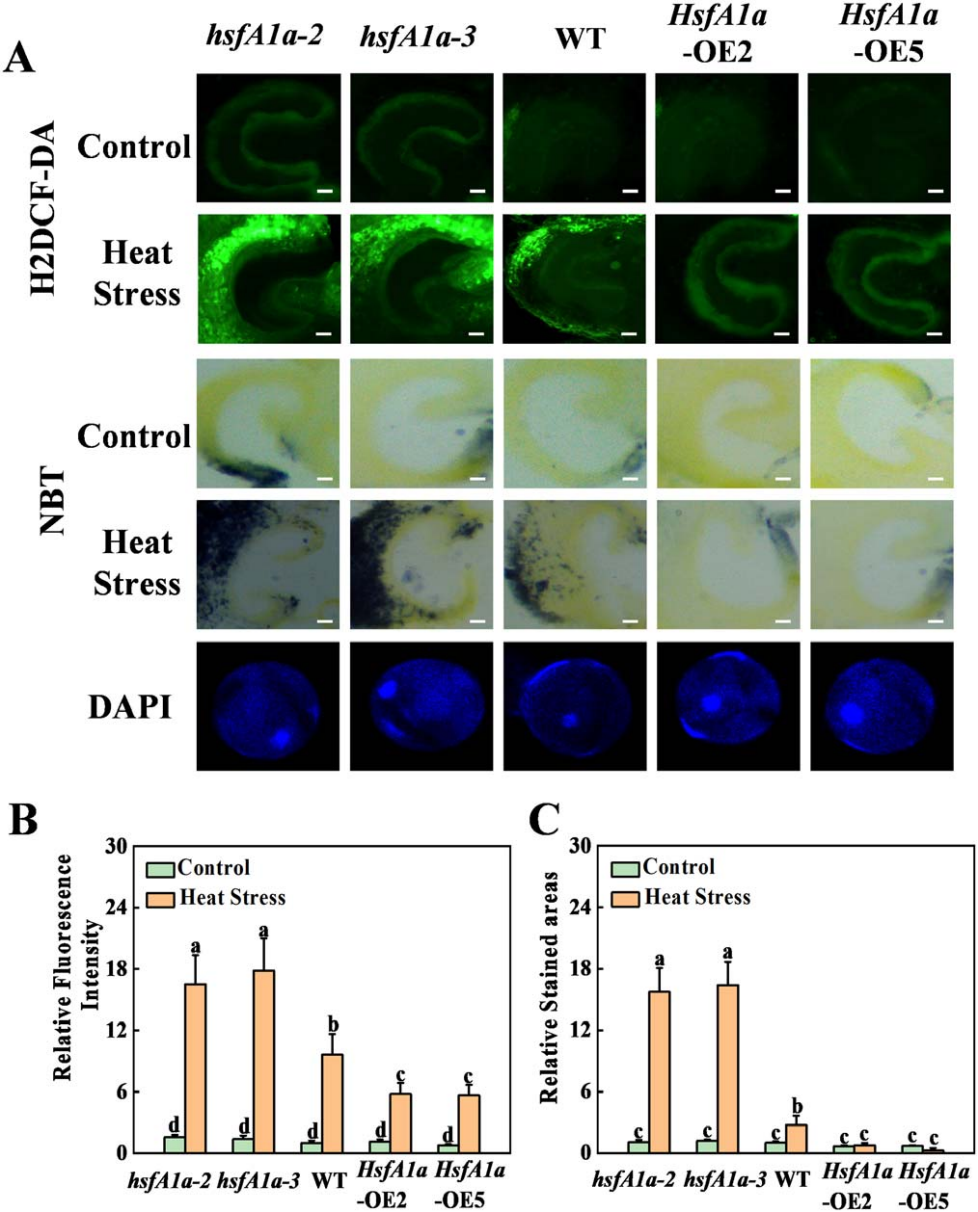
Loss or gain of function in HsfA1a affects heat-stress-induced ROS accumulation in tomato anthers. (A) Anther ROS detected by H_2_DCF-DA staining in cross-sections of anthers of *hsfA1a* mutants, WT, and *HsfA1a*-overexpressing (OE) plants. The content of superoxide (O_2_^•−^) in cross-sections of *hsfA1a*, WT, and *HsfA1a*-OE anthers was determined using NBT staining. Anther developmental stage was confirmed using DAPI staining of pollen grains. Scale bar: 100 μm for anthers, 10 μm for pollen. (B, C) Relative ROS fluorescence signal intensity (B) and relative area ratio of NBT staining (C) in cross-sections of anthers of *hsfA1a*, WT, and *HsfA1a*-OE plants. Means marked with the same letter in Tukey’s test indicate a non-significant difference at *P* < .05. The results were derived from quality analysis of ROS and O_2_^•−^ accumulation in anthers isolated from 30 individual flowers for each genotype and treatment. *hsfA1a*-2 and *hsfA1a*-3, two lines of *hsfA1a* mutants; *HsfA1a*-OE2 and *HsfA1a*-OE5, two lines of *HsfA1a*-OE plants.

Abiotic stresses such as heat can lead to an imbalance in the redox system of plant cells, resulting in oxidative stress [31]. To determine the effect of HsfA1a on the regulation of the redox system in tomato anthers under heat stress, we used 2′,7′-dichlorodihydrofluorescein diacetate (H_2_DCF-DA) and nitroblue tetrazolium (NBT) staining to assay the accumulation of ROS and superoxide (O_2_^•−^) in anthers of different genotypes at the polarized microspore stage. The H2DCF-DA-stained ROS and NBT-stained O_2_^•−^ in anthers kept signals at low levels under normal growth conditions in all genotypes. (Fig. 3). Heat stress significantly induced ROS accumulation in anthers of WT plants. Importantly, the ROS levels were increased by 71.8 and 85.4% and the O_2_^•−^ levels were increased by 5.5-fold and 6.0-fold in two lines of *hsfA1a* mutants, while the ROS levels were decreased by 39.5 and 40.9% and the O_2_^•−^ levels were decreased by 65.5 and 72.2% in two lines of *HsfA1a*-OE plants compared with WT (Fig. 3). The results suggested that HsfA1a regulates the scavenging ability of heat-induced ROS, thereby maintaining the homeostasis of the cellular redox system in anthers.

**Figure 4:**
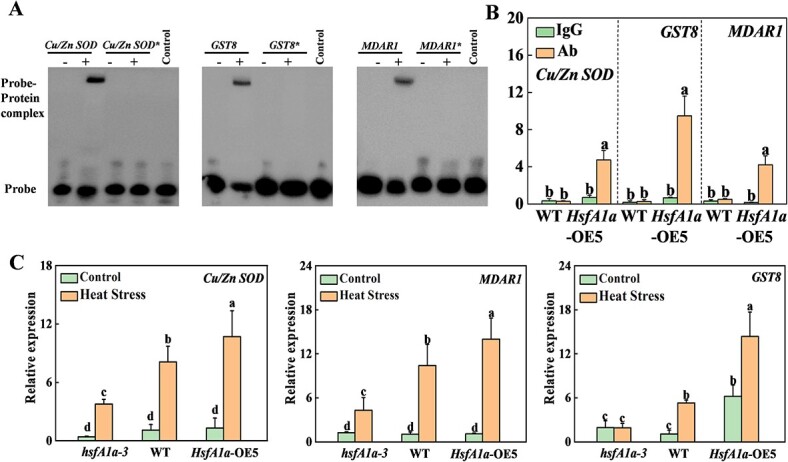
HsfA1a transcriptionally regulates *Cu/Zn SOD*, *GST8*, and *MDAR1* genes in anthers under heat stress. (A) EMSA showing binding of HsfA1a to HSE sequences of the promoters of *Cu/Zn SOD*, *GST8*, and *MDAR1.* Recombinant HsfA1a was used in DNA binding assays to *Cu*/*Zn SOD*, *GST8*, *MDAR1*, and their HSE motif sequences as probes. His was used as the negative control. The asterisk (*) represents a mutated probe. All probes are listed in Supplementary Data Table S4. (B) Binding of HsfA1a to the *Cu/Zn SOD*, *GST8*, and *MDAR1* promoters in *35S-HsfA1a-HA*-overexpressing (*HsfA1a*-OE) plants was analyzed using ChIP–qPCR. WT and *HsfA1a*-OE plants at flowering stage were subjected to heat-stress treatment, anther samples were taken, and input chromatin was isolated from them. Anti-HA antibody was applied for immunoprecipitation of HsfA1a–chromatin complexes. Control reactions used mouse IgG. qRT–PCR was used to quantify input and ChIP DNA samples with specific primers for the promoters of *Cu/Zn SOD*, *GST8*, and *MDAR1* genes. The percentages of input DNA are displayed as ChIP results. (C) Relative expression of *Cu/Zn SOD*, *GST8*, and *MDAR1* genes in anthers of *hsfA1a*, WT, and *HsfA1a*-OE plants under control or heat stress. Means with the same letter in Tukey’s test indicate a non-significant difference at *P* < .05. Similar results were obtained in three independent experiments. *hsfA1a*-3, one line of *hsfA1a* mutants; *HsfA1a*-OE5, one line of *HsfA1a* OE plants.

Antioxidant defense mechanism-mediated ROS scavenging is essential for pollen resistance and fertility under abiotic stresses [32]. To determine the possible regulation of antioxidant capacity by HsfA1a, we examined the 1.5-kb sequence located upstream of the predicted transcription start site of 25 tomato antioxidant-related genes. Among them, the promoters of eight antioxidant enzyme genes contained the HSE element (GAANNTTC) (Supplementary Data Fig. S3A). Then, an electrophoretic mobility assay (EMSA) was performed to detect the binding of HsfA1a to the promoters of these eight antioxidant enzyme genes *in vitro*. As shown in Fig. 4A and Supplementary Data Fig. S3B, signals of probe–protein complexes were detected using biotin-labeled specific probes. Five genes (*GSTL3*, *GST8*, *GSTU25*, *MDAR1*, *Cu/Zn SOD*) could be bound by HsfA1a protein *in vitro*. When the HSE motifs of these five gene promoters were mutated, the HsfA1a binding probe signals were completely lost (Fig. 4A, Supplementary Data Fig. S3B). We performed chromatin immunoprecipitation (ChIP)–qPCR assays to further determine the binding of tomato HsfA1a to the promoters of these five antioxidant enzyme genes in anthers under high temperature. The results are shown in Fig. 4B. The promoter sequences of *Cu/Zn SOD*, *GST8*, and *MDAR1* genes were significantly enriched with anti-hemagglutinin (HA) Monoclonal antibody. 3HA-labeled HsfA1a transgene products were immunoprecipitated the in anthers of *HsfA1a*-OE5 plants after heat stress, but they were not enriched in WT plants. By contrast, the promoters of these three genes could not be pulled down by the IgG control antibody in anthers of both WT and *HsfA1a*-OE5 plants (Fig. 4B). Therefore, HsfA1a directly binds to the promoters of *Cu/Zn-SOD*, *GST8*, and *MDAR1* genes under heat stress. Furthermore, we verified the expression of *Cu/Zn SOD*, *GST8*, and *MDAR1* in anthers after heat stress using qRT–PCR analysis. The expression trends were consistent between the qRT–PCR results and quantitative RNA-seq data (Figs 2D and 4C). The three genes were expressed at similar levels in *hsfA1a* mutants, WT, and *HsfA1a*-OE plants under the control condition (Fig. 4C). However, heat-induced expression of these three genes was compromised in anthers of *hsfA1a* mutants, whereas they were significantly upregulated in WT plants and further amplified in *HsfA1a*-OE plants (Fig. 4C). Interestingly, the activities of SOD and POD were decreased in anthers of WT plants and were further compromised in anthers of *hsfA1a* mutants after heat stress exposure. Importantly, SOD, POD, GST, and monodehydroascorbate reductase (MDAR) maintained higher activity in the anthers of *HsfA1a*-OE plants under high temperature compared with WT (Supplementary Data Fig. S4). Thus, these findings indicated that HsfA1a could directly regulate the transcription of *Cu/Zn SOD*, *GST8*, *MDAR1* genes and improve the antioxidant capacity of anthers under heat stress.

### HsfA1a mediates heat shock protein expression and accumulation in anthers under heat stress

Studies have demonstrated that many Hsfs participate in the regulation of the pollen HSR under heat stress [1]. As HsfA1a is a major regulator of the HSR network, the different levels of HsfA1a may affect the expression of other Hsfs. We used RT–qPCR to detect the expression trends of *Hsf* genes in anthers of different genotypes after heat stress (Supplementary Data Fig. S5), and the results were consistent with the RNA-seq results (Fig. 2D). The expression levels of *HsfA1a*, *HsfA1b*, *HsfA1c*, *HsfA2*, *HsfA3*, and *HsfB2* in anthers of WT and *HsfA1a*-OE5 plants were induced by heat stress and were significantly upregulated in *HsfA1a*-OE5 compared with WT. In contrast, in *HsfA1a* mutant anthers, the expression of *HSFA1a* and *HsfA1b* and *HsfA1c* was not induced by heat stress. The expression of *HsfA2* and *HsfB2* was upregulated after heat stress, but remained significantly lower than that of WT (Supplementary Data Fig. S5). One possible implication of this manifestation is that Hsfs may have partial functional overlap and the expression of other *HsfA*s may be suppressed in the presence of high *HsfA1a* expression.

**Figure 5:**
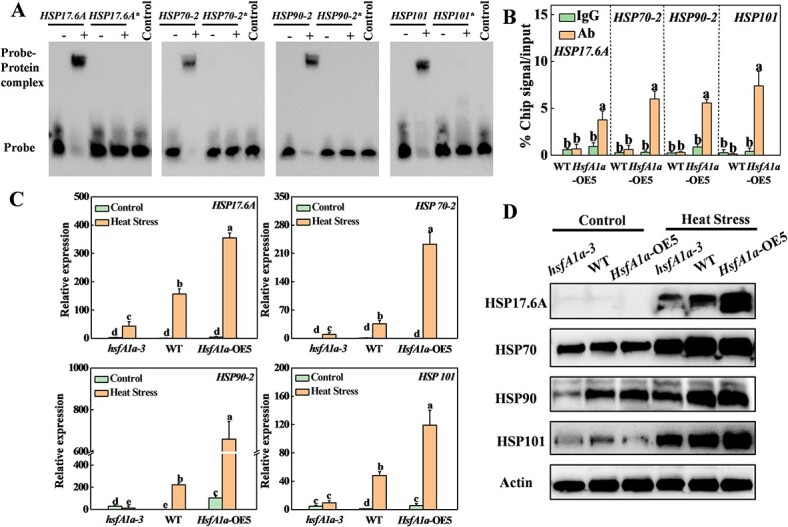
HsfA1a regulates expression and protein accumulation of HSPs in tomato anthers under heat stress. (A) EMSA analysis showing binding of HsfA1a to HSE sequences of the promoters of *HSP17.6A*, *HSP70-2*, *HSP90-2*, and *HSP101*. Recombinant HsfA1a was applied for DNA binding assays to *HSP17.6A*, *HSP70-2*, *HSP90-2*, and *HSP101* and their HSE motif sequences as the probes. His is displayed as the negative control. Asterisks (*) represent mutated probes. All probes are listed in Supplementary Data Table S4. (B) ChIP–qPCR was used to validate HsfA1a binding to the promoters of *HSP17.6A*, *HSP70-2*, *HSP90-2*, and *HSP101* in *35S-HsfA1a-HA*-overexpressing (*HsfA1a*-OE) plants. WT and *HsfA1a*-OE plants were subjected to heat stress at flowering stage, anther samples were taken and input chromatin was isolated from them. Anti-HA antibody immunoprecipitation was used to epitope-tag the HsfA1a–chromatin complex. Mouse IgG treatment was used as a control reaction. qRT–PCR was used to quantify input and ChIP DNA samples with specific primers for the promoters of *HSP17.6A*, *HSP70-2*, *HSP90-2*, and *HSP101* genes. The percentages of input DNA are displayed as ChIP results. (C) Relative expression of *HSP17.6A*, *HSP70-2*, *HSP90-2*, and *HSP101* genes in anthers of *hsfA1a*, WT, and *HsfA1a*-OE plants under control or heat stress. (D) Accumulation of HSP17.6, HSP70, HSP90, and HSP101 proteins in anthers of *hsfA1a*, WT, and *HsfA1a*-OE plants under control or heat stress. Actin was displayed as a loading control in western blotting. Means with the same letter in Tukey’s test indicate a non-significant difference at *P* < .05. Similar results were obtained in three independent experiments. *hsfA1a*-3, one line of *hsfA1a* mutants; *HsfA1a*-OE5, one line of *HsfA1a*-OE plants.

HsfAs regulate the expression of heat shock proteins on a different scale after heat stress exposure [7]. To analyze the function of tomato HsfA1a in the regulation of HSPs in anthers after heat stress, we examined the 1.5-kb sequence located upstream of the predicted transcription start site of 16 tomato HSPs genes (Fig. 2D). Among them, the promoters of eight HSP genes contained HSE elements (Supplementary Data Fig. S6A). Therefore, we performed EMSA analysis and found that HsfA1a protein could directly bind to the promoters of six HSP genes (*HSP17.6A*, *HSP70-1*, *HSP70-2*, *HSP90-1*, *HSP90-2*, *HSP101*) *in vitro*, whereas when the HSE motifs of the promoters of these six genes were mutated, binding to HsfA1a was completely lost (Fig. 5A, Supplementary Data Fig. S6B). Meanwhile, we performed ChIP–qPCR to examine whether tomato HsfA1a binds to the promoters of these HSP genes in heat-stressed anthers. The promoter sequences of *HSP17.6A*, *HSP70-2*, *HSP90-2*, and *HSP101* were significantly enriched by anti-HA antibodies (Fig. 5B). 3HA-tagged HsfA1a transgene products were immunoprecipitated in anthers of *HsfA1a*-OE5 plants after heat stress, but not in WT. In contrast, the promoter DNA fragments of these four genes failed to be pulled down by the IgG control antibody (Fig. 5B). Thus, HsfA1a binds to four HSP gene promoters under high temperature. In addition, we performed qRT–PCR analysis to verify the expression of these four genes (*HSP17.6A*, *HSP70-2*, *HSP90-2*, *HSP101*) in heat-stressed anthers, and the results were similar to the expression trends of RNA-seq data (Figs 5C and 2D). Under normal temperature conditions, the transcript abundances of the four HSP genes were consistent in different genotypes (Fig. 5C). The expression of the four HSP genes in anthers of *hsfA1a* mutants was significantly lower than in those of WT plants after heat stress, while *HsfA1a*-OE plants showed higher expression levels compared with WT (Fig. 5C). We also applied western blotting to determine the accumulation of HSP proteins in anthers after heat stress (Fig. 5D). Under normal conditions, HSP70 and HSP90 proteins accumulated in anthers of different genotypes, while HSP17.6 and HSP101 were less accumulated. Under heat stress, WT and *HsfA1a*-OE plants had more HSP protein accumulation compared with the *hsfA1a* mutant (Fig. 5D). The findings of these studies suggested that HsfA1a can directly regulate the transcription levels of *HSP17.6A*, *HSP70-2*, *HSP90-2*, and *HSP101* genes and the accumulation of corresponding HSP proteins, and then improve the protection ability of proteins in tomato anthers under heat stress.

### HsfA1a promotes degradation of ubiquitinated proteins by autophagy and 26S proteasome

**Figure 6:**
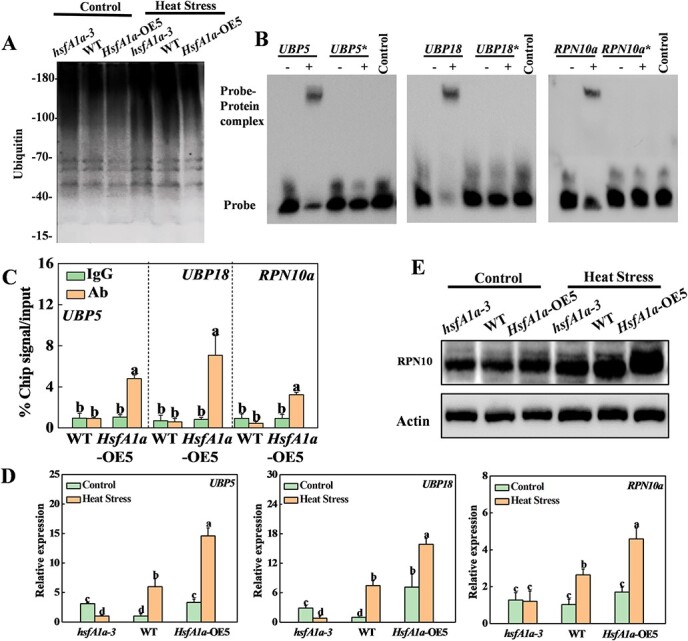
HsfA1a transcriptionally regulates ubiquitin- and proteasome-related genes in tomato anthers under heat stress. (A) Accumulation of ubiquitination for total proteins. Ubiquitinated proteins were subjected to SDS–PAGE and analyzed with an anti-ubiquitin monoclonal antibody in anthers of *hsfA1a*, WT, and *HsfA1a*-overexpressing (OE) plants. (B) EMSA analysis showing binding of HsfA1a to HSE sequences of the promoters of *UBP5*, *UBP18*, and *RPN10a*. Recombinant HsfA1a was used in DNA assays of binding to *UBP5*, *UBP18*, and *RPN10a*, and their HSE motif mutated sequences as the probes. Asterisks (*) represent mutated probes. All probes are listed in Supplementary Data Table S2. His is the negative control. (C) ChIP–qPCR was used to validate HsfA1a binding to the *UBP5*, *UBP18*, and *RPN10a* promoters in *35S-HsfA1a-HA*-OE (*HsfA1a*-OE) plants. Anther samples of WT and *HsfA1a*-OE plants at flowering stage after heat stress were obtained, and input chromatin was isolated from them. Anti-HA antibody immunoprecipitation was used to epitope-tag the HsfA1a–chromatin complex. Mouse IgG treatment was used as a control reaction. qRT–PCR was used to quantify input and ChIP DNA samples with specific primers for the promoters of the *UBP5*, *UBP18*, and *RPN10a* genes. The percentages of input DNA are displayed as ChIP results. (D) Relative expression of *UBP5*, *UBP18*, and *RPN10a* genes in anthers of *hsfA1a*, WT, and *HsfA1a*-OE plants under the control condition or heat stress. (E) Accumulation of RPN10 proteins in anthers of *hsfA1a*, WT, and *HsfA1a*-OE plants under the control condition or heat stress. Actin was used as a loading control. Means with the same letter in Tukey’s test indicate a non-significant difference at *P* < .05. Similar results were obtained in three independent experiments. *hsfA1a*-3, one line of *hsfA1a* mutants; *HsfA1a*-OE5, one line of *HsfA1a*-OE plants.

Heat stress induces abundant denatured proteins that are easily aggregated and ubiquitinated in plants [19, 33]. To confirm whether HsfA1a is involved in the regulation of denatured protein degradation, the anther proteins of different genotypes were separated and analyzed for ubiquitination using western blotting. Under control conditions, the level of ubiquitinated proteins was increased in *hsfA1a* mutants, but was similar in *HsfA1a*-OE plants when compared with WT plants (Fig. 6A). The accumulation of ubiquitinated proteins was increased in anthers of WT plants and further increased in anthers of *hsfA1a* mutants under heat stress (Fig. 6A). Importantly, overexpression of *HsfA1a* alleviated the accumulation of ubiquitinated proteins in anthers after heat stress exposure (Fig. 6A). Thus, HsfA1a promoted the degradation of ubiquitinated proteins in heat-stress-exposed tomato anthers.

**Figure 7:**
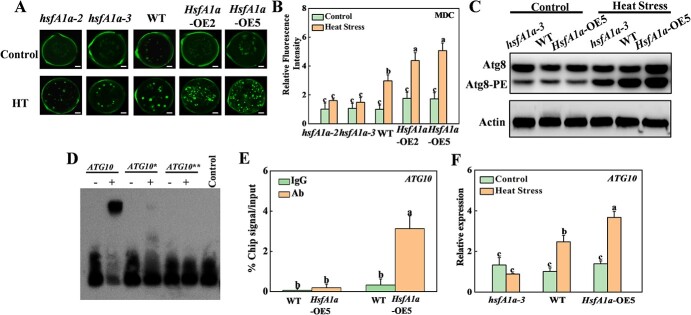
HsfA1a regulates autophagy and *ATG10* expression under heat stress in tomato anthers. (A) MDC-stained autophagosomes in tomato pollens. Scale bar: 10 μm. MDC-stained spots are represented as green signals. (B) Relative fluorescence intensity of MDC staining of pollen. More than 100 MDC-stained pollens were tested for each genotype and treatment. (C) Atg8 protein levels in tomato anthers. Atg8-PE represents the non-lipidated form of Atg8. Actin was used as a loading control. (D) EMSA analysis showing the binding of HsfA1a to the *ATG10* promoter. Recombinant HsfA1a was applied to bind with *ATG10* and its HSE motif mutated sequence as the probes. Asterisks represents ATG10 mutated probes. All probes are listed in Supplementary Data Table S4. His was a negative control. (E) ChIP–qPCR was used to validate HsfA1a binding to the *ATG10* promoter in *35S-HsfA1a-HA*-OE (*HsfA1a*-OE) plants. Anther samples of WT and *HsfA1a*-OE plants at flowering stage after heat stress were obtained, and input chromatin was isolated from them. Anti-HA antibody immunoprecipitation was used to epitope-tag the HsfA1a–chromatin complex. Mouse IgG treatment was used as a control reaction. qRT–PCR was used to quantify input and ChIP–DNA samples with specific primers for the *ATG10* promoters. The percentages of input DNA are displayed as ChIP results. (F) Relative expression of *ATG10* gene in anthers of *hsfA1a*, WT, and *HsfA1a*-OE plants under control or heat stress. Means with the same letter in Tukey’s test indicate a non-significant difference at *P* < .05. Similar results were obtained in three independent experiments. *hsfA1a-2* and *hsfA1a*-3, two lines of *hsfA1a* mutants; *HsfA1a*-OE2 and *HsfA1a*-OE5, two lines of *HsfA1a*-OE plants.

Ubiquitinated proteins are degraded by the UPS and autophagy pathways [20, 21]. To examine whether HsfA1a regulates the UPS pathway to degrade ubiquitinated proteins in the anther response to heat stress, we examined the upstream sequences of the predicted transcription start sites of 22 tomato ubiquitin-related genes and 11 proteasome-related genes and found that the promoters of 12 genes contained HSE elements (Supplementary Data Figs S7B and S8A). EMSA analysis showed the binding of HsfA1a to the promoters of seven genes (*UBP18*, *UBP5*, *UBA1*, *E3-like*, *RPN5a*, *RPN5b*, *RPN10a*) *in vitro*. The binding signals to HsfA1a were completely lost when the HSE motifs of these seven genes were mutated (Fig. 6B, Supplementary Data Figs S7B and S8A). We used ChIP–qPCR to determine whether HsfA1a could bind to the promoters of these genes in anthers under heat stress. The results demonstrated that the promoter sequences of *UBP5*, *UBP18*, and *RPN10a* were enriched by anti-HA antibodies (Fig. 6C). The 3HA-tagged HsfA1a transgene product was immunoprecipitated in *HsfA1a*-OE5 plants after heat stress, but they was not enriched in WT plants (Fig. 6C). Meanwhile, the IgG control antibody could not pull down the promoter DNA fragments of these three genes in the anthers of WT and *HsfA1a*-OE5 plants (Fig. 6C). Thus, HsfA1a binds to the promoters of the *UBP5*, *UBP18*, and *RPN10a* genes under heat stress. At the same time, qRT–PCR analysis also confirmed that the expression trends of these three genes (*UBP5*, *UBP18*, and *RPN10a*) under heat stress were consistent with the RNA-seq data (Figs 6D and 2D). The transcription levels of the *UBP5* and *UBP18* genes in *hsfA1a* mutants and *HsfA1a*-OE plants were slightly higher than those in WT under normal temperature conditions (Fig. 6D), while the expression of *RPN10a* was similar in plants of different genotypes. However, compared with WT, these three genes had lower expression in *hsfA1a* mutants and had higher expression levels in *HsfA1a*-OE plants after heat stress (Fig. 6D). Furthermore, we examined the protein accumulation of RPN10 in heat-stressed anthers using western blotting (Fig. 6E). As shown in Fig. 6E, the accumulation of RPN10 in anthers of plants of different genotypes at normal temperature already reached a high level. The accumulation of RPN10 protein in *hsfA1a* mutant anthers after heat stress was lower than in WT, while *HsfA1a*-OE plants accumulated more compared with WT (Fig. 6E). The results above demonstrated that HsfA1a directly regulates the transcription of the *UBP5*, *UBP18*, and *RPN10a* genes, thereby affecting the protein ubiquitination level and the degradation ability of ubiquitinated proteins in tomato anthers under heat stress.

To further investigate whether HsfA1a regulates autophagy in response to heat stress, we conducted monodansylcadaverine (MDC) staining to detect autophagy activity in the tomato pollens. As shown in Fig. 7A, only slight punctate fluorescent signals were observed in the pollens of all *hsfA1a* mutants, WT, and *HsfA1a*-OE plants. However, the punctate fluorescent signals were increased 3-fold in the pollens of WT plants under 3 h heat treatment (Fig. 7B). However, heat-induced autophagic signals were impaired in *hsfA1a* pollens and increased in *HsfA1a*-OE pollens, compared with WT (Fig. 7B).

Atg8 has been used extensively to monitor autophagosomes. To detect autophagy activation in tomato anthers, we analyzed Atg8-phosphatidylethanol-amine (Atg8-PE) conjugates using western blotting and detected it with anti-Atg8a antibody as a marker for autophagy activation [34]. The Atg8-PE bands were similar in anthers of all *hsfA1a* mutants, WT, and *HsfA1a*-OE plants under normal temperature, but Atg8-PE bands were markedly enriched after heat stress (Fig. 7C). Interestingly, the heat-induced Atg8-PE band was compromised in *hsfA1a* mutants but more pronounced in *HsfA1a*-OE plants (Fig. 7C). In our previous study, we reported that HsfA1a can induce autophagy by transcriptional regulation of *ATG10* and *ATG18f* genes under drought in tomato plants [5]. Then, we detected whether HsfA1a could transcriptionally regulate *ATG10* and *ATG18f* in tomato pollens under heat stress. EMSA analysis showed that HsfA1a could bind to HSE elements of two ATG genes *in vitro*, and the binding signals disappeared after mutation of the HSE motif (Fig. 7D). The subsequent ChIP–qPCR assays showed that the promoter sequence of the *ATG10* gene was significantly enriched by anti-HA antibody, and 3HA-tagged HsfA1a transgenic products were immunoprecipitated in anthers of *HsfA1a*-OE5 plants after heat stress, but not enriched in WT plants (Fig. 7E). We further verified the expression of *ATG10* gene in anthers after heat stress using qRT–PCR analysis, and the expression trend was consistent with the qRT–PCR results and quantitative RNA-seq data (Figs 7F and 2D). Under normal growth conditions, *ATG10* has basically the same expression level in anthers of different genotypes. The expression of *ATG10* in anthers of WT and *HsfA1a*-OE plants was induced by heat stress and was significantly higher in *HsfA1a*-OE plants than in WT. Thus, the above results suggested that HsfA1a can directly regulate the transcription of the *ATG10* gene, thereby increasing autophagosome formation in pollen and the level of autophagy in tomato anthers under heat stress.

## Discussion

Heat stress can induce pollen HSR, leading to transcriptome, proteome, and metabolome reprogramming, which restores metabolism in pollen to maintain cellular homeostasis [1]. Hsfs are basic transcription factors in all organisms and are the basic elements of the HSR. Many studies have shown that Hsfs play central roles during plant growth and development and the response to abiotic stresses [30]. The present work demonstrates the importance of HsfA1a in tomato pollen development and heat tolerance.

The antioxidant enzyme system is essential for regulating ROS production and the development of pollen and anthers [32]. However, the redox balance would be disrupted under heat stress, and the cellular damage caused by the massive accumulation of ROS will further affect the development of anthers and pollen, thus requiring elaborate control mechanisms to deal with potential oxidative damage without interfering with cell signaling [31, 35]. Phytohormones such as abscisic acid, jasmonic acid, and salicylic acid have been reported to improve pollen fertility under heat stress by enhancing the scavenging ability of antioxidant systems in rice spikelets [32]. Meanwhile, many transcription factors are closely involved in the development and stress resistance of pollens and anthers by regulating the redox system. For instance, the accumulation of excess ROS in the stamens of the *Arabidopsis myb21* mutant resulted in defective stamen development [36]. Overexpression of *MYB Important for Drought Response1* (*MID1*) in rice anthers increases *POD* and *SOD* transcript levels, thereby enhancing ROS scavenging ability to enhance drought tolerance [37]. Our study showed that high temperature resulted in massive ROS accumulation in tomato anthers, but this could be rescued by *HsfA1a* overexpression. As a major positive regulator of HSR, HsfA1a could transcriptionally regulate the expression of the *GST8*, *MDAR1*, and *Cu*/*Zn SOD* genes, thereby enhancing the activity of antioxidant enzymes and detoxifying ROS in tomato anthers under heat stress. These studies suggested that HsfA1a is closely related to the balanced regulation of ROS production and scavenging in pollen under heat stress.

Abiotic stresses often lead to damage of proteins in pollen and anther cells. In addition to being repaired, some are degraded through the UPS and autophagy pathways to avoid aggregation and their toxic effects on cells [21, 22]. The damaged proteins produced by stresses such as high temperature need to be modified by ubiquitination before they are degraded through the UPS pathway [15, 16, 19]. Studies have confirmed that many genes encoding E2 and E3 ligases are stress-inducible and could modulate plant development and responses to various abiotic stresses [38]. Cold stress promotes RING-type E3 ligase (HOS1)-dependent proteasome degradation of the flowering transcription factor Constans (CO) and causes earlier flowering in *hos1* mutant plants [39]. In addition, recycling ubiquitin is essential for maintaining the monoubiquitin pool and facilitating protein degradation [22, 40]. *Arabidopsis AtUBP3/4* is involved in pollen tube development. The pollen of the *ubp3 ubp4* mutant is incapable of undergoing mitosis II and exhibits pollen germination defects [25]. Herein, we found that a large number of ubiquitin- and proteolysis-related genes were significantly upregulated in heat-stressed tomato anthers, and demonstrated that the expression of ubiquitin-related genes (*UBP5* and *UBP18*) at high temperature was transcriptionally regulated by HSFA1a, which ultimately affected the accumulation of ubiquitinated proteins in anthers (Fig. 6). The 26S proteasome utilizes its own protease activity to degrade target proteins, regulating plant developmental processes and responses to environmental stimuli at multiple levels [21]. Defects in 26S proteasome function lead to a series of plant developmental problems. The *rpt2a* mutant exhibits impaired leaf, root, trichome, and pollen development and prolonged flowering, and the RPT2 subunit is a component of the 26S proteasome [24]. RPN10 is a subunit of the 26S proteasome that serves as a ubiquitin receptor subunit through ubiquitin interactions. Our results indicated that HsfA1a could transcriptionally regulate the expression of *RPN10a* in anthers under heat stress and further induce the accumulation of RPN10 protein. Moreover, some regulatory particle subunits are transcriptionally regulated or phosphorylated under stress conditions, and some 20S regulatory particle subunits may be translationally modified under stress conditions, leading to altered proteasome assembly and activity [21]. Our RNA-seq data showed that 34% of protein phosphorylation-related and 31% of methylation-related genes were induced by heat stress in WT tomato anthers, but not in *hsfA1a* mutants (Fig. 2C, Supplementary Data Table S2). Thus, loss of HsfA1a function may severely affect post-transcriptional and translational modification processes, which in turn affect normal heat stress-related responses in tomato anthers.

**Figure 8:**
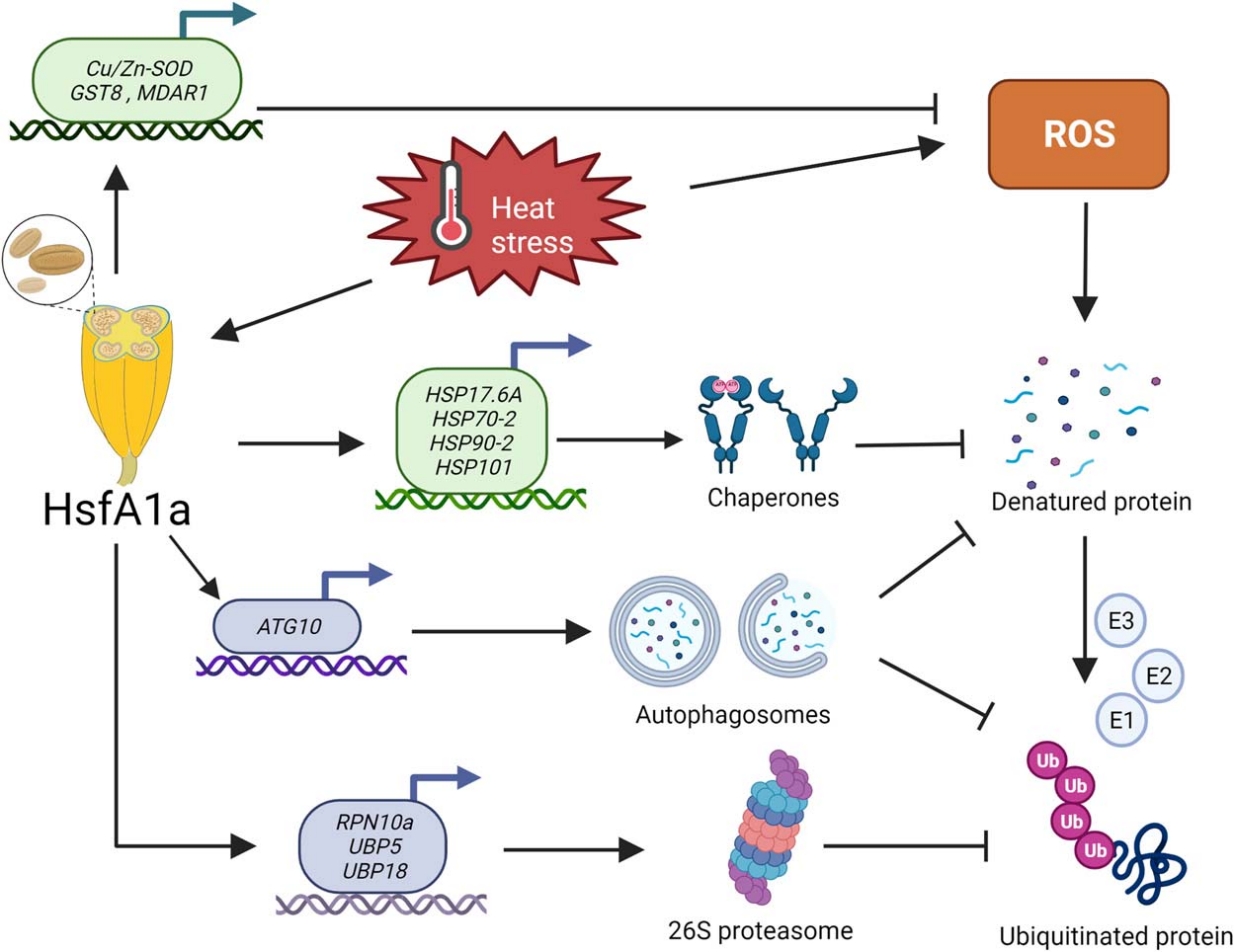
Proposed model for HsfA1a-mediated pollen thermotolerance in tomato plants under heat stress. HsfA1a can induce the expression of antioxidant enzyme genes (*Cu/Zn-SOD*, *GST8*, and *MDAR1*) to enhance the scavenging ability of ROS and reduce oxidative damage in anthers. Furthermore, HsfA1a mediates heat-induced denatured protein repair and degradation. On the one hand, HsfA1a regulates the expression of *HSP17.6A*, *HSP70-2*, *HSP90-2*, and *HSP101* and increases the accumulation of HSPs to maintain the conformation of proteins and refold heat-damaged proteins. On the other hand, HsfA1 promotes UPS and autophagy pathways to degrade denatured and ubiquitinated proteins by transcriptional regulation of the key genes (*UBP5*, *UBP18*, *RPN10a*, and *ATG10*) in both pathways.

The UPS has a size limit on degraded molecules, and some oligomeric and aggregated proteins caused by stresses such as high temperature are too large to pass through the narrow proteasome entry channel [21]. In contrast, autophagy can degrade protein aggregates, intact protein complexes, and even organelles [20]. Previous studies confirmed that high temperature can significantly induce the expression of ATGs and the accumulation of autophagosomes in tomato and pepper (*Capsicum annuum* L.) plants [33, 41]. Herein, autophagy in pollens and anthers was also induced by heat stress. Our transcriptome data showed that the transcription levels of multiple ATGs, such as *ATG10*, *ATG18d*, *ATG18g*, and *ATG18h*, were induced at high temperature. Moreover, the number of punctate fluorescent signals of autophagosomes in tomato plant pollen was significantly increased after heat stress. In addition, studies have shown that ATGs can be induced by transcriptional regulation under different stresses. Brassinosteroid signaling can regulate ATG expression and autophagosome formation, which is important for coping with nitrogen starvation in tomato [34]. Under drought stress, *ethylene response factor 5* (*ERF5*) transcriptionally regulates the expression of *ATG8d* and *ATG18h* and affects autophagy [42]. In our previous study, we also reported that HsfA1a transcriptionally regulated *ATG10* and *ATG18f* under drought stress [5]. However, in heat-stressed anthers, only *ATG10* could be transcriptionally regulated by HsfA1a. Meanwhile, we detected the accumulation of Atg8-PE protein in tomato anthers after heat stress, suggesting a potential role of autophagy in the regulation of heat tolerance in pollen and anthers.

In conclusion, we provide a comprehensive genetic and molecular analysis of tomato HsfA1a enhancing pollen heat stress resistance by upregulating antioxidant capacity, protein repair and degradation (Fig. 8). We demonstrate that HsfA1a can enhance pollen fertility by maintaining redox balance and protein homeostasis under heat stress. On the one hand, HsfA1a mediates heat-induced ROS scavenging through transcriptional regulation of the corresponding antioxidant enzymes. On the other hand, HsfA1a induces the accumulation and protein degradation pathways of HSPs under heat stress. Denatured proteins can be refolded and re-stabilized by HSP, and protein aggregates are degraded by the UPS or autophagy pathway under heat stress. Therefore, HsfA1a is involved in an effective protection for pollen under heat stress to maintain normal metabolic activities and cell homeostasis, finally improving pollen vigor and germination under heat stress. Furthermore, the role of epigenetic regulation in heat-induced transcriptional responses has received increasing attention. Hsfs may participate in the regulation of phosphorylation and methylation modifications. This study systematically elucidates the mechanism by which HsfA1a regulates tomato pollen heat tolerance, providing important information for refining the HSR regulatory network and potential applications.

## Materials and methods

### Plant materials and growth conditions

*Solanum lycopersicum* L. cv ‘Ailsa Craig’ was used in this study. We sowed seeds in trays with vermiculite and peat mixture (1:2/v:v). Three-week-old seedlings were transferred to plastic pots containing the same substrate. The conditions in the growth room were as follows: photoperiod was 12 hours/12 hours (light/dark), temperature was 25/20°C (day/night), and light intensity was 600 μmol m^−2^ s^−1^. For heat treatment, 8-week-old plants at the flowering stage were placed in a growth chamber at 39°C for 3 hours, and control plants were placed at 25°C for 3 hours. Six replicates were used in each treatment.

### Vector construction and generation of transgenic plants

To obtain *hsfA1a* mutants, a CRISPR-associated 9 (Cas9) vector was constructed according to the previously reported method [43]. The target sgRNA sequence for *HsfA1a* (GGAAGCCATCATGGAGGGAA) was designed using the CRISPR rgenome tools (http://www.rgenome.net/). Single-guide RNA (sgRNA) was annealed into a double strand and inserted into the AtU6-sgRNA-AtUBQ-Cas9 vector. The recombinant vector was confirmed by sequencing. After digestion with KpnI/HindIII, the purified target fragment was combined with the same cloning sites of the pCAMBIA1301 vector. The resulting plasmid was then transformed into *Agrobacterium tumefaciens* strain EHA105 and used to infect the cotyledons of the tomato ‘Ailsa Craig’ seedlings. Two independent Cas9-free T2 *hsfA1a* lines carrying mutations were identified for further experiments (Supplementary Data Fig. S1A). Two independent homozygous transgenic plants overexpressing *HsfA1a* (*HsfA1a*-OE2 and *HsfA1a*-OE5) used for the experiments were constructed as previously described [5].

### Phenotype analysis

Eight-week-old (flowering stage) tomato plants were transferred to the growth chamber at 39°C for 3 hours. Mature pollen grains were separated from anthers for viability and germination rate determination according to the methods described previously [35]. The pollen nuclei were stained with DAPI [6] and visualized by fluorescence microscopy (Leica, Germany).

### Transcriptome analysis

Total RNA was obtained and purified with TRIzol reagent from 0.5-g anther samples (Sigma–Aldrich, T9424). The RNA-seq experiment was performed in accordance with the manufacturer’s protocol, and the data were analyzed by LC Biotech (http://www.lc-bio.com/). Poly(A) RNA was obtained using magnetic beads linked to poly-T oligonucleotides and then fragmented into small 300-bp (±50-bp) pieces and reverse-transcribed to generate cDNA. Sequencing was then conducted with an Illumina Hiseq 4000 according to the supplier’s recommended methods. Expression of all transcripts were estimated using StringTie [44] and edgeR [45] after generating the final transcriptome. The differentially expressed mRNAs and genes were selected having | log_2_(FPKM) | >1 and *P* <. 05.

### Histochemical assays of reactive oxygen species

H_2_DCF-DA (Sigma–Aldrich, D6883) staining was used to detect ROS accumulation in tomato anthers according to previously described methods [35]. The O_2_^•−^ in anthers was detected by NBT (Aladdin, N274358) staining according to previously described methods [35]. Histological staining and quantitative fluorescence analysis were performed with Image J (https://imagej.nih.gov/ij/) as described previously [46].

### Antioxidant enzyme activity assay

Anther tissue (0.3 g) was homogenized with 3 ml of cold enzyme buffer and centrifuged at 12 000 g for 10 minutes. The resulting supernatant was then used to measure enzymatic activity with a UV-2410PC spectrophotometer (Shimadzu, Japan). The activities of SOD, POD, CAT, and ascorbate peroxidase were measured according to the previously described protocol [47]. MDAR [48] and GST [49] enzymatic activities were measured following previous protocols.

### MDC staining

In order to detect autophagosomes in pollen, MDC staining was performed as previously described [34]. The samples were washed gently with 100 mM PBS buffer followed by the addition of 10 mM MDC dye (Sigma–Aldrich, D4008) and subsequently stained for 30 minutes in the dark. Finally, samples were again washed with PBS buffer and analyzed with a confocal laser scanning microscope (Nikon, Japan).

### Total RNA extraction and gene expression analyses

Total RNA of tomato anthers was obtained using the Easy Plant RNA Kit (Easy-do, DR0406050) and reversed to cDNA using the ReverTra Ace qPCR RT Kit (Toyobo, FSQ-301). qRT–PCR used SYBR Green PCR Master Mix (Takara, RR420A) and the LightCycler^®^ 480 II Real-Time PCR detection system (Roche). Tomato *Actin* and *SAND* acted as internal controls, and the corresponding primer sequences used are shown in Supplementary Data Table S5. A previously reported method [50] was used for data analysis.

### Recombinant protein and electrophoretic mobility assay analyses

Recombinant protein preparation of tomato HsfA1a has been described [5]. The 1.5-kb sequences located upstream of genomic sequence for analysis of HSE-binding elements were downloaded directly from the website https://solgenomics.net. The probes were biotin-labeled and annealed using a Biotin 30 End DNA Labeling Kit (Pierce, 89 818). EMSA experiments were conducted using the LightShift Chemiluminescence EMSA Kit (ThermoFisher, 20148). The probe sequences used in the experiment are shown in Supplementary Data Table S4.

### Chromatin immunoprecipitation assay

The ChIP assay was performed with the EpiQuik™ Plant ChIP Kit (Epigentek, P-2014). After 3 hours of heat stress treatment at 39°C, 1.5 g of anther tissues was obtained from HA-tagged HsfA1a-OE and WT plants and the input chromatin was extracted. For ChIP experiments, HA antibody (ThermoFisher, 26183) was used for chromatin immunoprecipitation, and anti-mouse IgG (Millipore, AP124) was used as a negative control. The primers used in ChIP–qPCR analysis are specifically designed according to the target gene promoter, as shown in Supplementary Data Table S6.

### Protein extraction and western blotting

Total protein was extracted from 0.1-g tomato anther samples with 300 μl protein extraction buffer. SDS–PAGE of denatured proteins with a 13.5% gel containing 6 M urea was used to detect Atg8 [5]. Samples for detection of other proteins (HSPs and RPN10a) were separated by 10% SDS–PAGE. The ubiquitinated proteins were extracted and examined as described previously [19]. Western blot analysis was performed as described previously [5], and the corresponding antibodies used are shown in Supplementary Data Table S7. SuperSignal™ West Pico Chemiluminescent substrate (ThermoFisher, 34080) was used to detect the complexes on the blot.

### Statistical analysis

Five independent replicates for each determination were performed and statistical analysis was performed using SPSS statistical software (version 25.0, Chicago). Experimental data were analyzed using Tukey’s test at *P* < .05.

## Acknowledgements

This work was supported by the National Key Research and Development Program of China (2018YFD1000800), the National Natural Science Foundation of China (31922078 and 31872089), the Fundamental Research Funds for the Central Universities (226-2022-00122), and the Starry Night Science Fund of Zhejiang University Shanghai Institute for Advanced Study (SN-ZJU-SIAS-0011).

## Author contributions

D.-L.X. and J.Z. conceived this study and wrote the manuscript. D.-L.X., H.-M.H., C.-Y.Z., C.-X.L., M.K.K., and Z.-Y.Q. performed the experiments. D.-L.X. and J.Z. analyzed the data. All authors read and approved the manuscript.

## Data availability

The data supporting the findings of this study are available from the corresponding author (J.Z.) upon request.

## Conflicts of interest

No potential conflict of interest was reported by the authors.

## Supplementary Material

Web_Material_uhac163
