# Correction to: HsfA1a confers pollen thermotolerance through upregulating antioxidant capacity, protein repair, and degradation in *Solanum lycopersicum* L.

**DOI:** 10.1093/hr/uhae210

**Published:** 2022-07-22

This is a correction to: Dong-Ling Xie, Hua-Min Huang, Can-Yu Zhou, Chen-Xu Liu, Mukesh Kumar Kanwar, Zhen-Yu Qi, Jie Zhou, HsfA1a confers pollen thermotolerance through upregulating antioxidant capacity, protein repair, and degradation in *Solanum lycopersicum* L., *Horticulture Research*, Volume 9, 2022, https://doi.org/10.1093/hr/uhac163

In the originally published online version of this manuscript, there were errors within Figure 3A. During the manuscript preparation process, two correct images were inadvertently overwritten by redundant images due to an oversight. They are H_2_DCF-DA fluorescence image of WT under control (1^st^ row, panel 3) and NBT staining image of *hsfA1a-3* under control (3^rd^ row, panel 2).


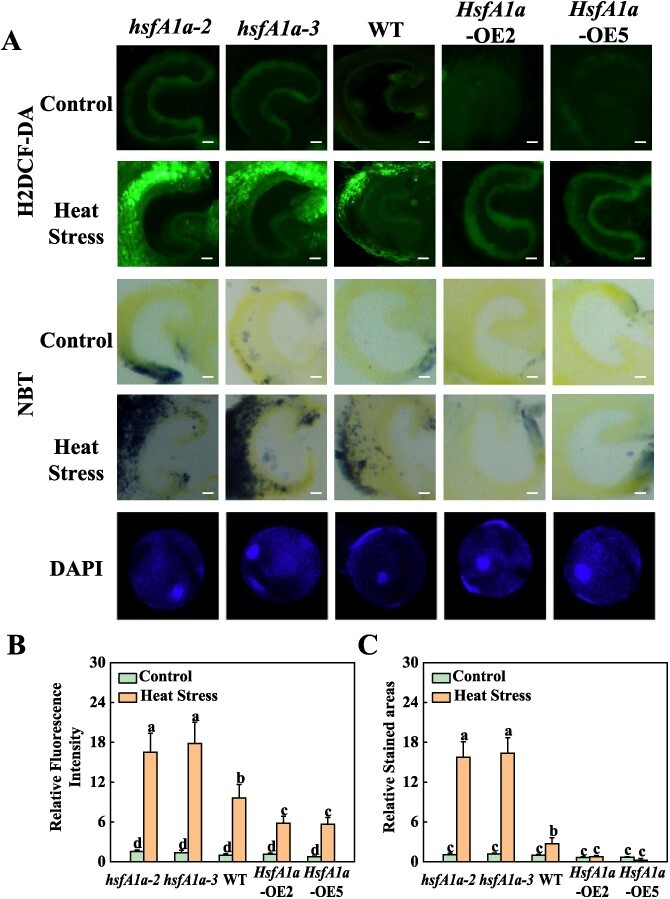
instead of:


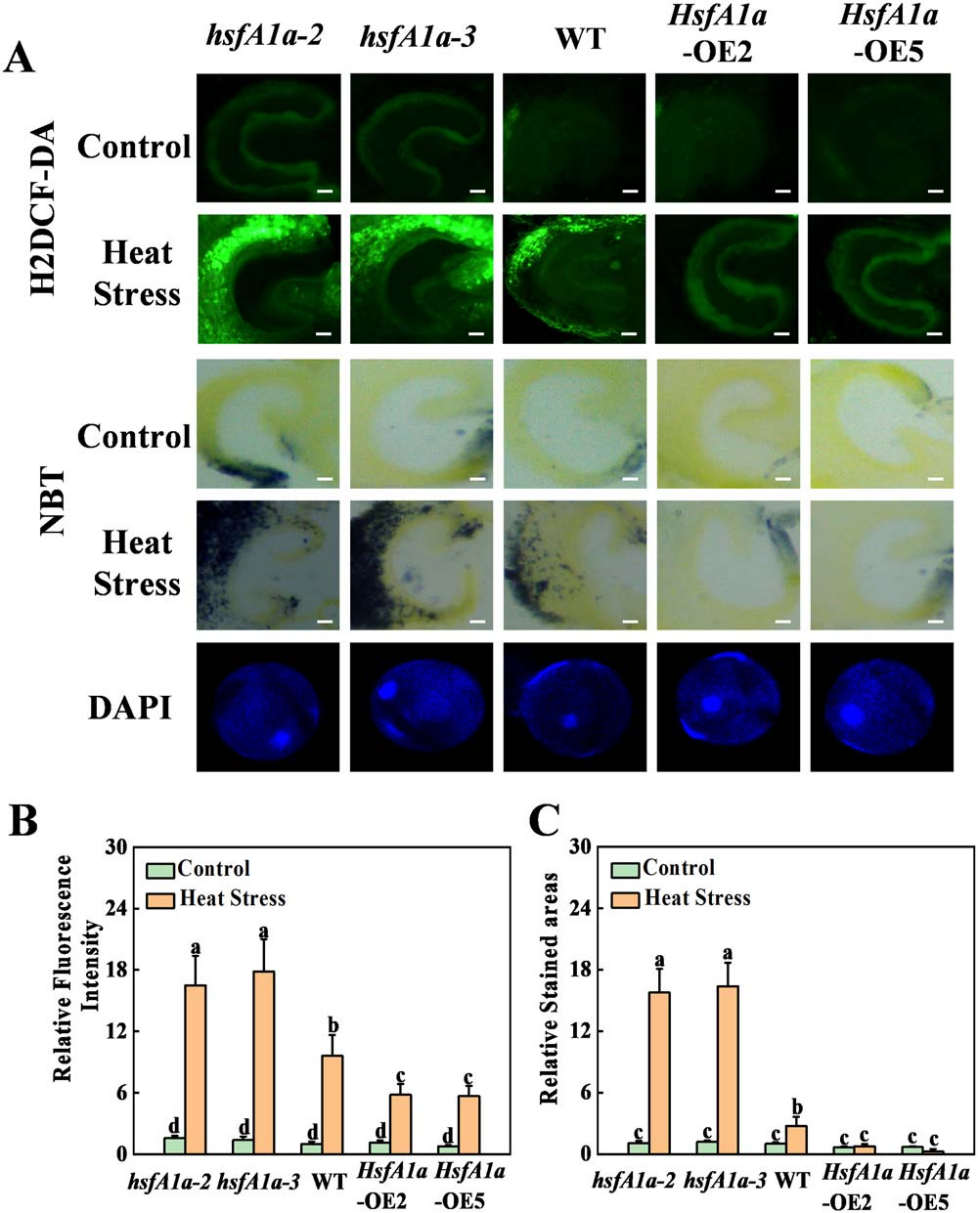
These corrections do not compromise the scientific integrity of the study’s conclusions.

This emendation has been outlined only in this correction notice to preserve theversion of record.

