# Supplementary material for: HsfA1a confers pollen thermotolerance through upregulating antioxidant capacity, protein repair, and degradation in Solanum lycopersicum L
Source: Hortic Res. 2022 Jul 22;9:uhac163. doi: 10.1093/hr/uhac163 (PMC9531336; doi:10.1093/hr/uhac163)
Supplement: Web_Material_uhac163 [file web_material_uhac163.zip › Supplementary Figures.pptx]

## Slide 1
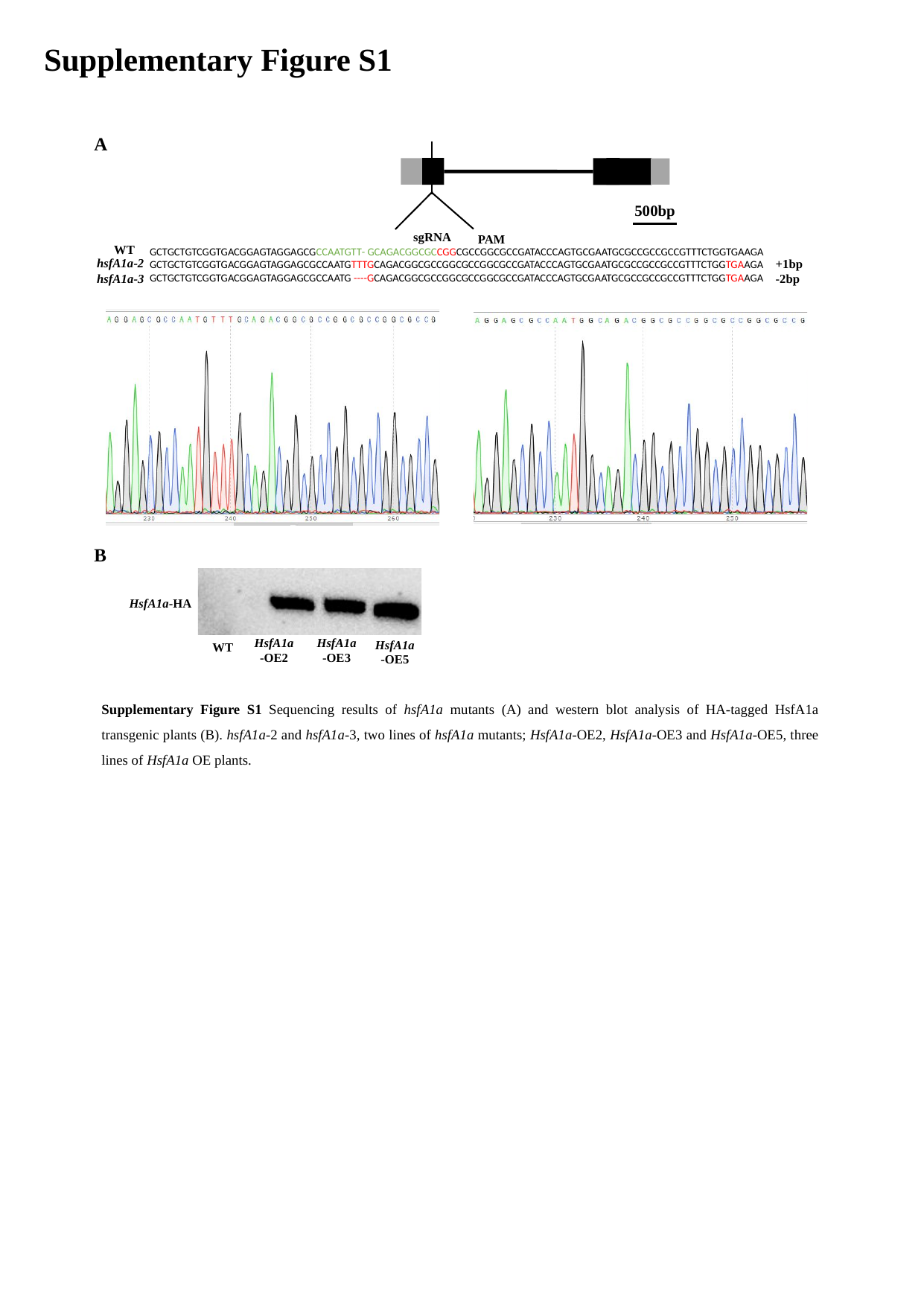

Supplementary Figure S1
A
500bp
sgRNA
PAM
WT
GCTGCTGTCGGTGACGGAGTAGGAGCGCCAATGTT- GCAGACGGCGCCGGCGCCGGCGCCGATACCCAGTGCGAATGCGCCGCCGCCGTTTCTGGTGAAGA
GCTGCTGTCGGTGACGGAGTAGGAGCGCCAATGTTTGCAGACGGCGCCGGCGCCGGCGCCGATACCCAGTGCGAATGCGCCGCCGCCGTTTCTGGTGAAGA
GCTGCTGTCGGTGACGGAGTAGGAGCGCCAATG ----GCAGACGGCGCCGGCGCCGGCGCCGATACCCAGTGCGAATGCGCCGCCGCCGTTTCTGGTGAAGA
hsfA1a-2
+1bp
-2bp
hsfA1a-3
B
HsfA1a
-OE3
HsfA1a
-OE2
WT
HsfA1a-HA
HsfA1a
-OE5
Supplementary Figure S1 Sequencing results of hsfA1a mutants (A) and western blot analysis of HA-tagged HsfA1a transgenic plants (B). hsfA1a-2 and hsfA1a-3, two lines of hsfA1a mutants; HsfA1a-OE2, HsfA1a-OE3 and HsfA1a-OE5, three lines of HsfA1a OE plants.

## Slide 2
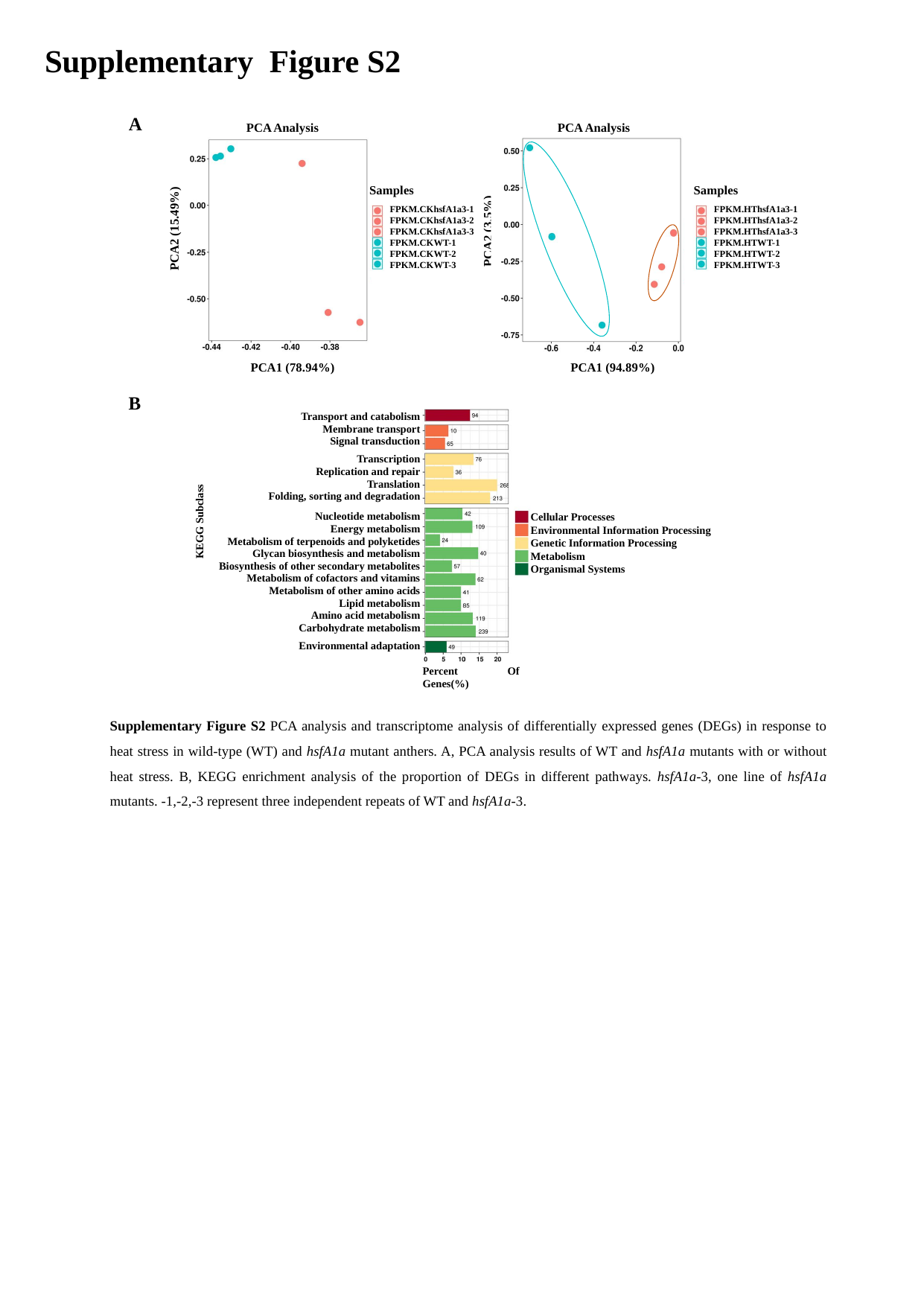

Supplementary Figure S2
A
PCA Analysis
Samples
FPKM.CKhsfA1a3-1
FPKM.CKhsfA1a3-2
FPKM.CKhsfA1a3-3
FPKM.CKWT-1
FPKM.CKWT-2
FPKM.CKWT-3
PCA2 (15.49%)
PCA1 (78.94%)
PCA Analysis
Samples
FPKM.HThsfA1a3-1
FPKM.HThsfA1a3-2
FPKM.HThsfA1a3-3
FPKM.HTWT-1
FPKM.HTWT-2
FPKM.HTWT-3
PCA1 (94.89%)
PCA2 (3.5%)
B
Transport and catabolism
Membrane transport
Signal transduction
Transcription
Replication and repair
Translation
Folding, sorting and degradation
Nucleotide metabolism
Energy metabolism
Metabolism of terpenoids and polyketides
Glycan biosynthesis and metabolism
Biosynthesis of other secondary metabolites
Metabolism of cofactors and vitamins
Metabolism of other amino acids
Lipid metabolism
Amino acid metabolism
Carbohydrate metabolism
Cellular Processes
Environmental Information Processing
Genetic Information Processing
Metabolism
Organismal Systems
KEGG Subclass
Environmental adaptation
Percent Of Genes(%)
Supplementary Figure S2 PCA analysis and transcriptome analysis of differentially expressed genes (DEGs) in response to heat stress in wild-type (WT) and hsfA1a mutant anthers. A, PCA analysis results of WT and hsfA1a mutants with or without heat stress. B, KEGG enrichment analysis of the proportion of DEGs in different pathways. hsfA1a-3, one line of hsfA1a mutants. -1,-2,-3 represent three independent repeats of WT and hsfA1a-3.

## Slide 3
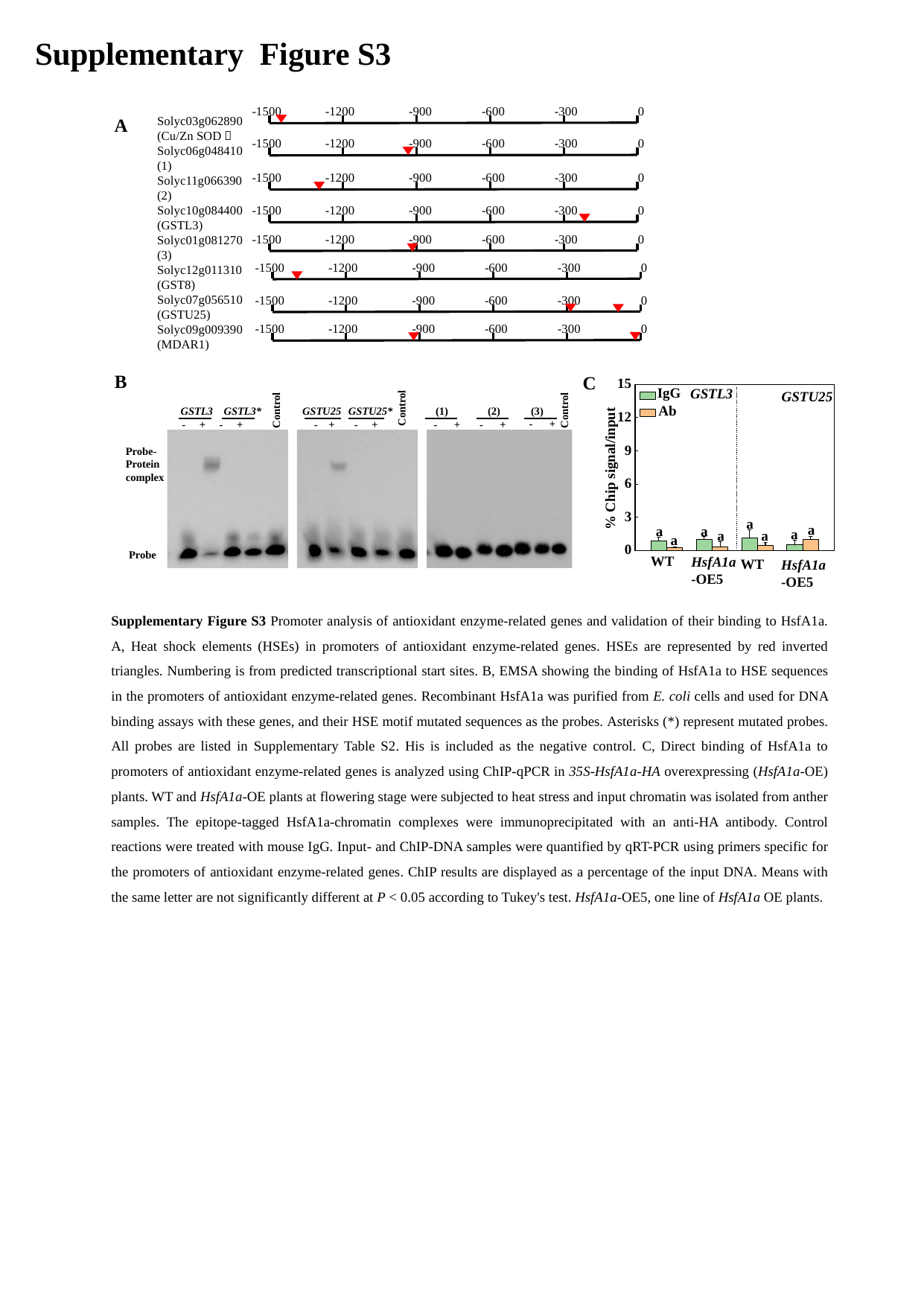

Supplementary Figure S3
-1500
-1200
-900
-600
-300
0
-1500
-1200
-900
-600
-300
0
-1500
-1200
-900
-600
-300
0
-1500
-1200
-900
-600
-300
0
-1500
-1200
-900
-600
-300
0
-1500
-1200
-900
-600
-300
0
-1500
-1200
-900
-600
-300
0
-1500
-1200
-900
-600
-300
0
Solyc03g062890
(Cu/Zn SOD）
Solyc06g048410
(1)
Solyc11g066390
(2)
Solyc10g084400
(GSTL3)
Solyc01g081270
(3)
Solyc12g011310
(GST8)
Solyc07g056510
(GSTU25)
Solyc09g009390
(MDAR1)
A
B
C
Control
Control
Control
GSTL3
GSTL3*
GSTU25
GSTU25*
 (1)
 (2)
 (3)
- +
- + - +
- + - +
- + - +
Probe-
Protein
complex
Probe
Supplementary Figure S3 Promoter analysis of antioxidant enzyme-related genes and validation of their binding to HsfA1a. A, Heat shock elements (HSEs) in promoters of antioxidant enzyme-related genes. HSEs are represented by red inverted triangles. Numbering is from predicted transcriptional start sites. B, EMSA showing the binding of HsfA1a to HSE sequences in the promoters of antioxidant enzyme-related genes. Recombinant HsfA1a was purified from E. coli cells and used for DNA binding assays with these genes, and their HSE motif mutated sequences as the probes. Asterisks (*) represent mutated probes. All probes are listed in Supplementary Table S2. His is included as the negative control. C, Direct binding of HsfA1a to promoters of antioxidant enzyme-related genes is analyzed using ChIP-qPCR in 35S-HsfA1a-HA overexpressing (HsfA1a-OE) plants. WT and HsfA1a-OE plants at flowering stage were subjected to heat stress and input chromatin was isolated from anther samples. The epitope-tagged HsfA1a-chromatin complexes were immunoprecipitated with an anti-HA antibody. Control reactions were treated with mouse IgG. Input- and ChIP-DNA samples were quantified by qRT-PCR using primers specific for the promoters of antioxidant enzyme-related genes. ChIP results are displayed as a percentage of the input DNA. Means with the same letter are not significantly different at P < 0.05 according to Tukey's test. HsfA1a-OE5, one line of HsfA1a OE plants.

## Slide 4
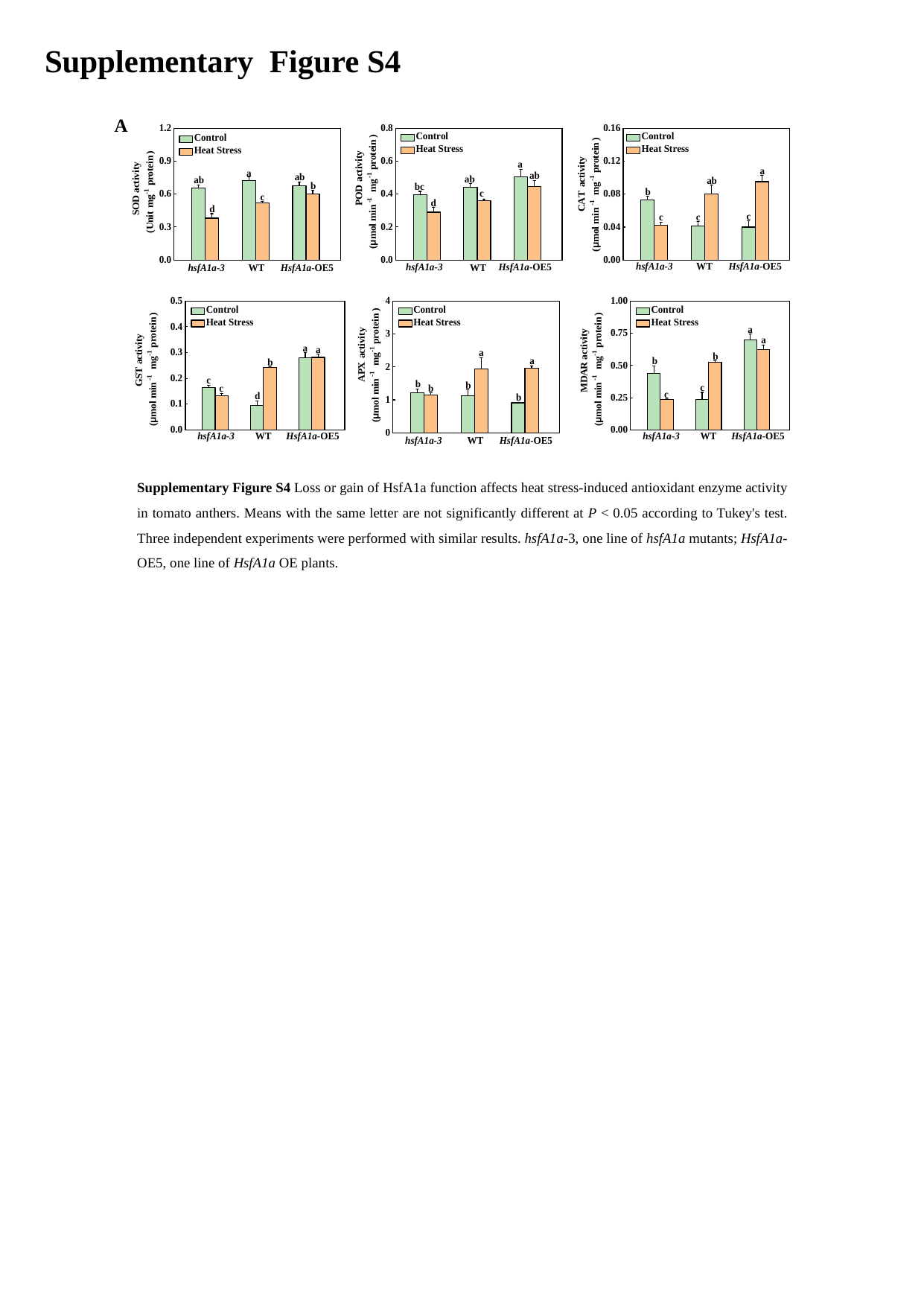

Supplementary Figure S4
A
Supplementary Figure S4 Loss or gain of HsfA1a function affects heat stress-induced antioxidant enzyme activity in tomato anthers. Means with the same letter are not significantly different at P < 0.05 according to Tukey's test. Three independent experiments were performed with similar results. hsfA1a-3, one line of hsfA1a mutants; HsfA1a-OE5, one line of HsfA1a OE plants.

## Slide 5
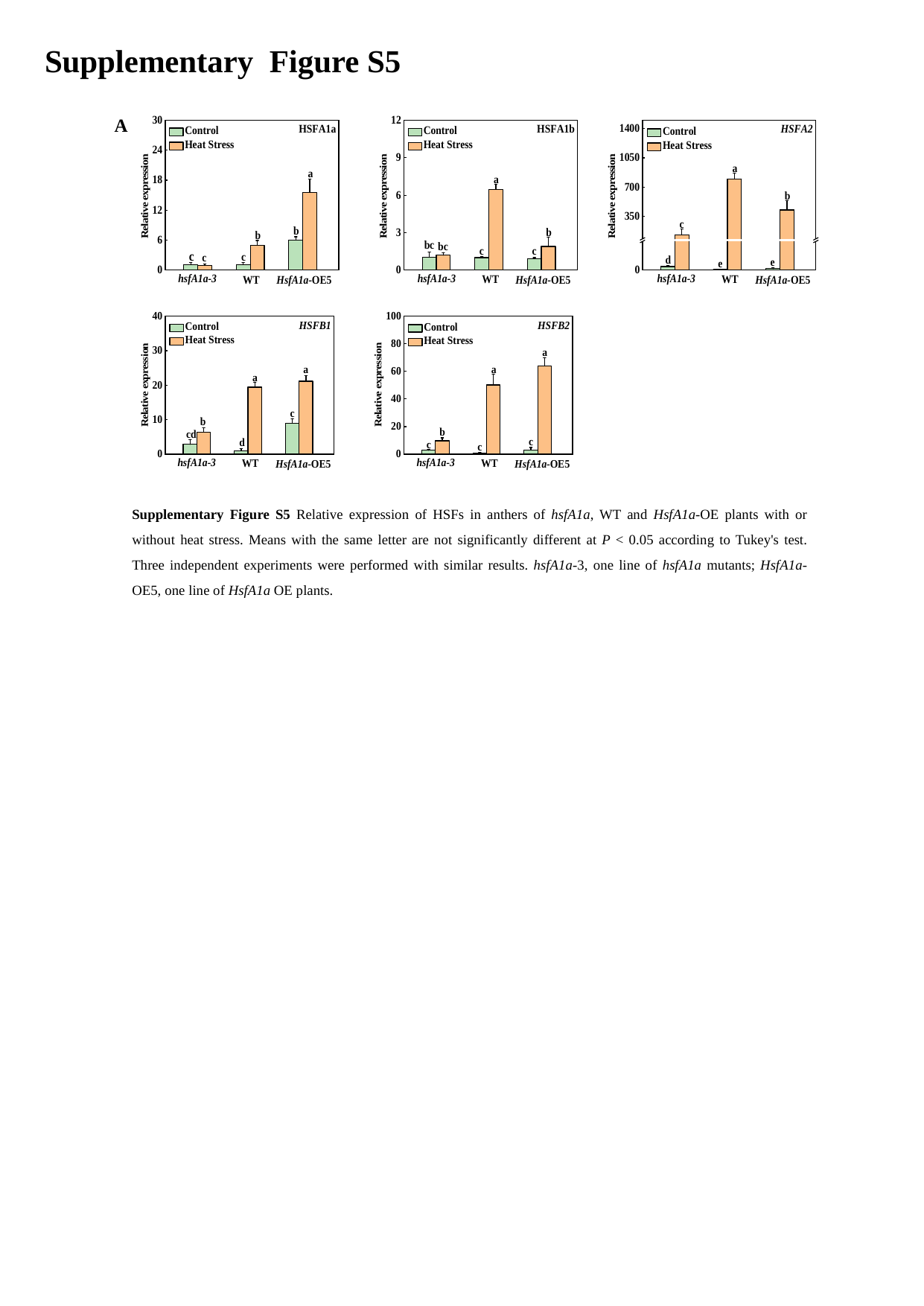

Supplementary Figure S5
A
Supplementary Figure S5 Relative expression of HSFs in anthers of hsfA1a, WT and HsfA1a-OE plants with or without heat stress. Means with the same letter are not significantly different at P < 0.05 according to Tukey's test. Three independent experiments were performed with similar results. hsfA1a-3, one line of hsfA1a mutants; HsfA1a-OE5, one line of HsfA1a OE plants.

## Slide 6
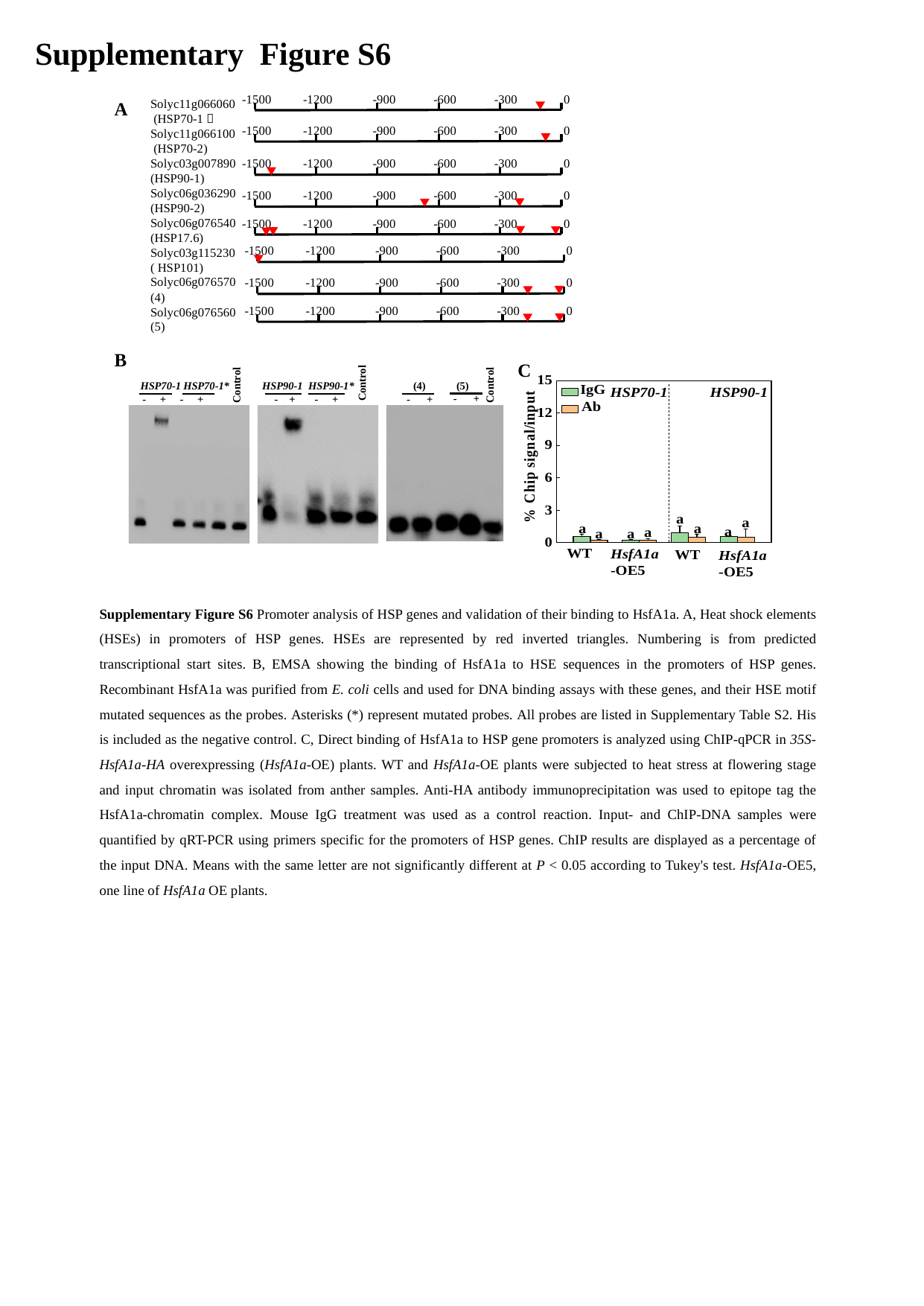

Supplementary Figure S6
-1500
-1200
-900
-600
-300
0
-1500
-1200
-900
-600
-300
0
-1500
-1200
-900
-600
-300
0
-1500
-1200
-900
-600
-300
0
-1500
-1200
-900
-600
-300
0
-1500
-1200
-900
-600
-300
0
-1500
-1200
-900
-600
-300
0
-1500
-1200
-900
-600
-300
0
Solyc11g066060
 (HSP70-1）
Solyc11g066100
 (HSP70-2)
Solyc03g007890
(HSP90-1)
Solyc06g036290
(HSP90-2)
Solyc06g076540
(HSP17.6)
Solyc03g115230
( HSP101)
Solyc06g076570
(4)
Solyc06g076560
(5)
A
B
C
Control
Control
Control
HSP70-1
HSP70-1*
HSP90-1
HSP90-1*
 (4)
 (5)
- +
- + - +
- + - +
 - +
Supplementary Figure S6 Promoter analysis of HSP genes and validation of their binding to HsfA1a. A, Heat shock elements (HSEs) in promoters of HSP genes. HSEs are represented by red inverted triangles. Numbering is from predicted transcriptional start sites. B, EMSA showing the binding of HsfA1a to HSE sequences in the promoters of HSP genes. Recombinant HsfA1a was purified from E. coli cells and used for DNA binding assays with these genes, and their HSE motif mutated sequences as the probes. Asterisks (*) represent mutated probes. All probes are listed in Supplementary Table S2. His is included as the negative control. C, Direct binding of HsfA1a to HSP gene promoters is analyzed using ChIP-qPCR in 35S-HsfA1a-HA overexpressing (HsfA1a-OE) plants. WT and HsfA1a-OE plants were subjected to heat stress at flowering stage and input chromatin was isolated from anther samples. Anti-HA antibody immunoprecipitation was used to epitope tag the HsfA1a-chromatin complex. Mouse IgG treatment was used as a control reaction. Input- and ChIP-DNA samples were quantified by qRT-PCR using primers specific for the promoters of HSP genes. ChIP results are displayed as a percentage of the input DNA. Means with the same letter are not significantly different at P < 0.05 according to Tukey's test. HsfA1a-OE5, one line of HsfA1a OE plants.

## Slide 7
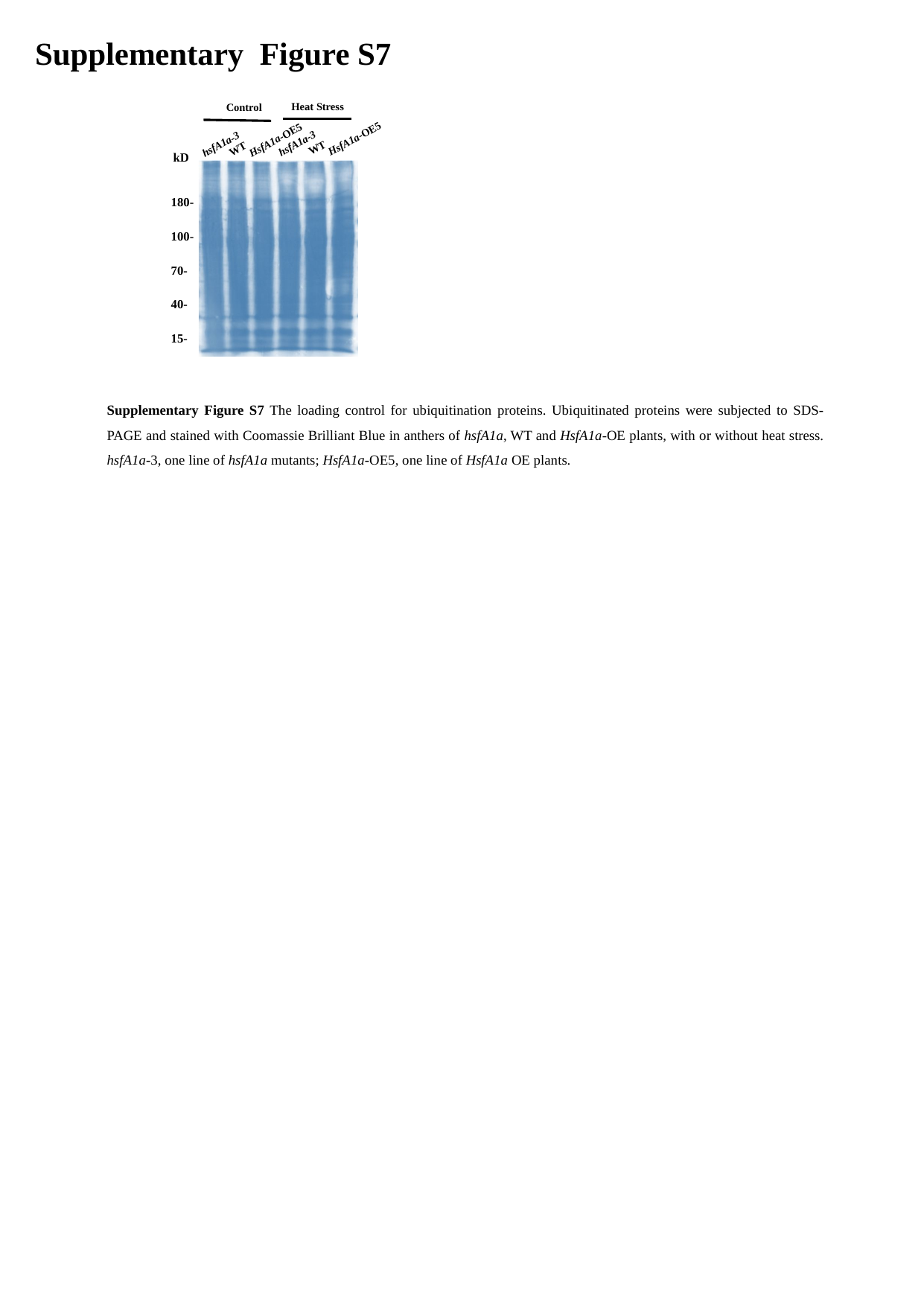

Supplementary Figure S7
Heat Stress
Control
hsfA1a-3
hsfA1a-3
WT
WT
HsfA1a-OE5
HsfA1a-OE5
kD
180-
100-
70-
40-
15-
Supplementary Figure S7 The loading control for ubiquitination proteins. Ubiquitinated proteins were subjected to SDS-PAGE and stained with Coomassie Brilliant Blue in anthers of hsfA1a, WT and HsfA1a-OE plants, with or without heat stress. hsfA1a-3, one line of hsfA1a mutants; HsfA1a-OE5, one line of HsfA1a OE plants.

## Slide 8
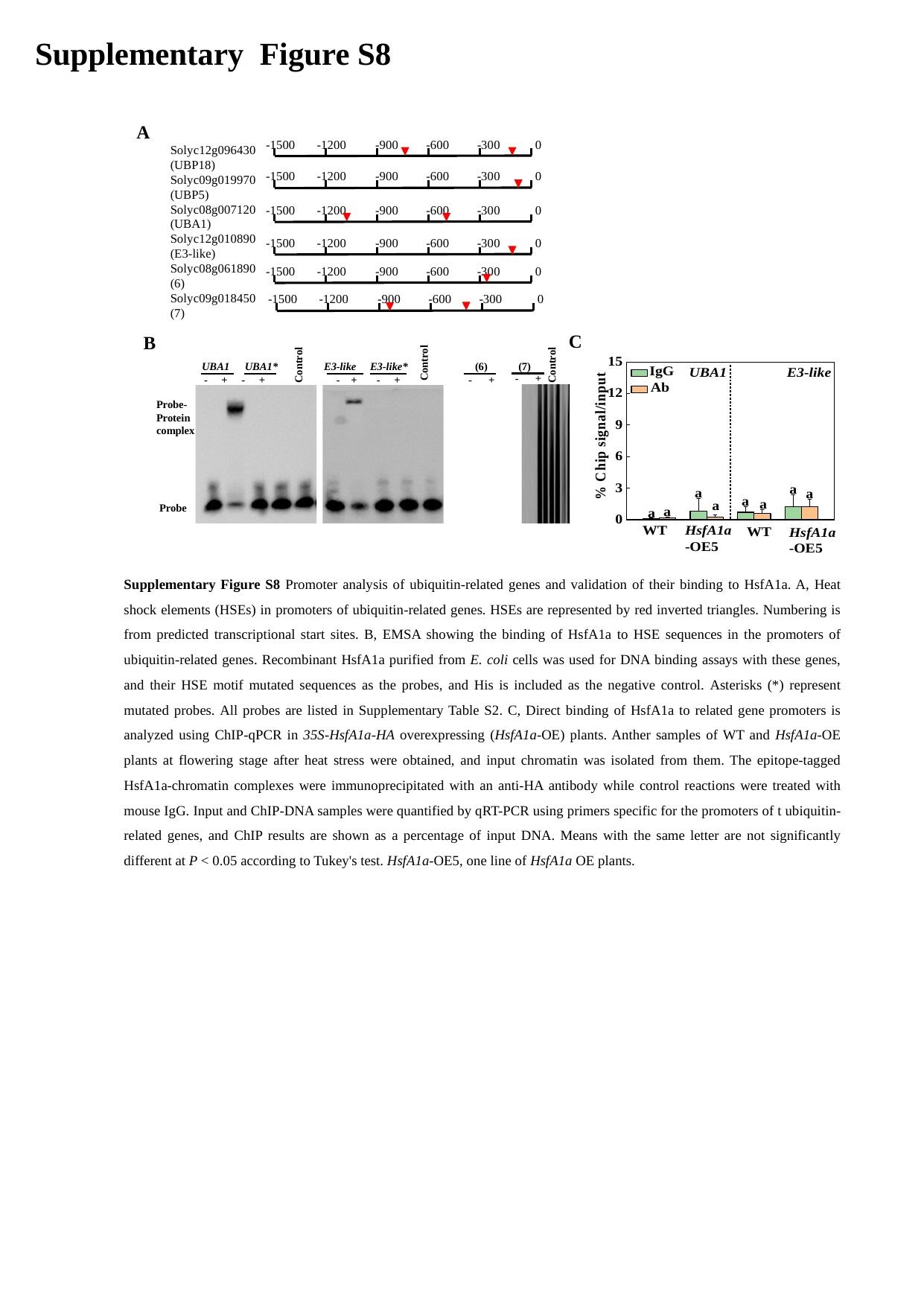

Supplementary Figure S8
A
-1500
-1200
-900
-600
-300
0
-1200
-900
-600
-300
0
-1500
-1200
-900
-600
-300
0
-1500
-1500
-1200
-900
-600
-300
0
-1200
-900
-600
-300
0
-1500
-1500
-1200
-900
-600
-300
0
Solyc12g096430
(UBP18)
Solyc09g019970
(UBP5)
Solyc08g007120
(UBA1)
Solyc12g010890
(E3-like)
Solyc08g061890
(6)
Solyc09g018450
(7)
C
B
Control
Control
Control
UBA1
UBA1*
E3-like
E3-like*
 (6)
 (7)
- +
- + - +
- + - +
 - +
Probe-
Protein
complex
Probe
Supplementary Figure S8 Promoter analysis of ubiquitin-related genes and validation of their binding to HsfA1a. A, Heat shock elements (HSEs) in promoters of ubiquitin-related genes. HSEs are represented by red inverted triangles. Numbering is from predicted transcriptional start sites. B, EMSA showing the binding of HsfA1a to HSE sequences in the promoters of ubiquitin-related genes. Recombinant HsfA1a purified from E. coli cells was used for DNA binding assays with these genes, and their HSE motif mutated sequences as the probes, and His is included as the negative control. Asterisks (*) represent mutated probes. All probes are listed in Supplementary Table S2. C, Direct binding of HsfA1a to related gene promoters is analyzed using ChIP-qPCR in 35S-HsfA1a-HA overexpressing (HsfA1a-OE) plants. Anther samples of WT and HsfA1a-OE plants at flowering stage after heat stress were obtained, and input chromatin was isolated from them. The epitope-tagged HsfA1a-chromatin complexes were immunoprecipitated with an anti-HA antibody while control reactions were treated with mouse IgG. Input and ChIP-DNA samples were quantified by qRT-PCR using primers specific for the promoters of t ubiquitin-related genes, and ChIP results are shown as a percentage of input DNA. Means with the same letter are not significantly different at P < 0.05 according to Tukey's test. HsfA1a-OE5, one line of HsfA1a OE plants.

## Slide 9
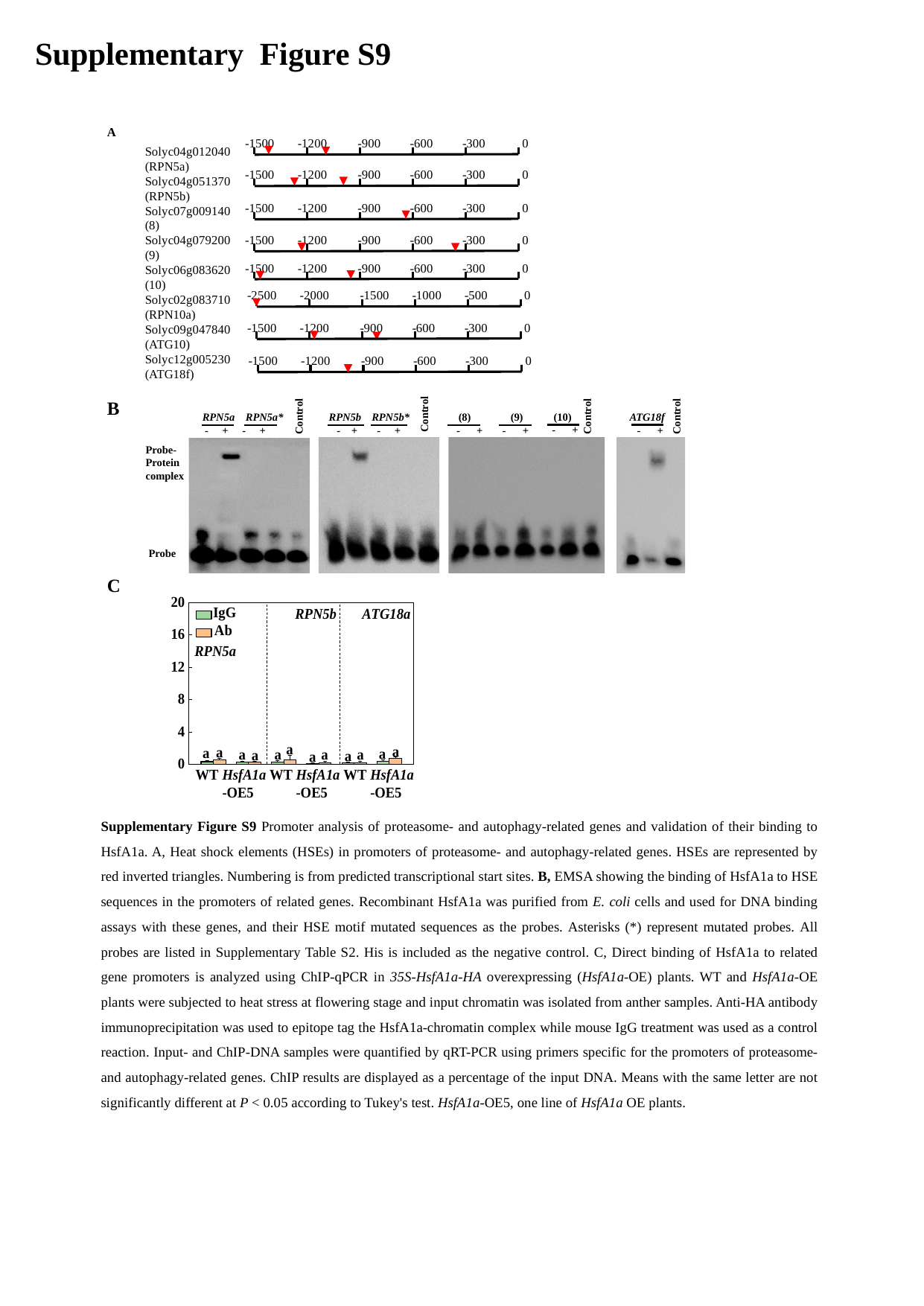

Supplementary Figure S9
A
Solyc04g012040
(RPN5a)
Solyc04g051370
(RPN5b)
Solyc07g009140
(8)
Solyc04g079200
(9)
Solyc06g083620
(10)
Solyc02g083710
(RPN10a)
Solyc09g047840
(ATG10)
Solyc12g005230
(ATG18f)
-1500
-1200
-900
-600
-300
0
-1500
-1200
-900
-600
-300
0
-1500
-1200
-900
-600
-300
0
-1500
-1200
-900
-600
-300
0
-1500
-1200
-900
-600
-300
0
-2500
-2000
-1500
-1000
-500
0
-1500
-1200
-900
-600
-300
0
-1500
-1200
-900
-600
-300
0
Control
Control
Control
Control
RPN5a
RPN5a*
RPN5b
RPN5b*
 (8)
 (9)
 (10)
ATG18f
- +
- + - +
- + - +
- + - +
- +
B
Probe-
Protein
complex
Probe
C
Supplementary Figure S9 Promoter analysis of proteasome- and autophagy-related genes and validation of their binding to HsfA1a. A, Heat shock elements (HSEs) in promoters of proteasome- and autophagy-related genes. HSEs are represented by red inverted triangles. Numbering is from predicted transcriptional start sites. B, EMSA showing the binding of HsfA1a to HSE sequences in the promoters of related genes. Recombinant HsfA1a was purified from E. coli cells and used for DNA binding assays with these genes, and their HSE motif mutated sequences as the probes. Asterisks (*) represent mutated probes. All probes are listed in Supplementary Table S2. His is included as the negative control. C, Direct binding of HsfA1a to related gene promoters is analyzed using ChIP-qPCR in 35S-HsfA1a-HA overexpressing (HsfA1a-OE) plants. WT and HsfA1a-OE plants were subjected to heat stress at flowering stage and input chromatin was isolated from anther samples. Anti-HA antibody immunoprecipitation was used to epitope tag the HsfA1a-chromatin complex while mouse IgG treatment was used as a control reaction. Input- and ChIP-DNA samples were quantified by qRT-PCR using primers specific for the promoters of proteasome- and autophagy-related genes. ChIP results are displayed as a percentage of the input DNA. Means with the same letter are not significantly different at P < 0.05 according to Tukey's test. HsfA1a-OE5, one line of HsfA1a OE plants.
